# Enhancing the Introduction and Scale Up of Self-Administered Injectable Contraception (DMPA-SC) in Health Systems (the EASIER Project): Protocol for Embedded Implementation Research

**DOI:** 10.2196/44222

**Published:** 2023-08-23

**Authors:** Colin Baynes, Petrus S Steyn, Kenneth Sherr, James Kiarie

**Affiliations:** 1 Department of Global Health University of Washington Seattle, WA United States; 2 UNDP/UNFPA/UNICEF/WHO/World Bank Special Programme of Research Development and Research Training in Human Reproduction Geneva Switzerland; 3 Department of Epidemiology University of Washington Seattle, WA United States; 4 Department of Industrial and Systems Engineering University of Washington Seattle, WA United States

**Keywords:** contraception, depo-medroxyprogesterone acetate subcutaneous, DMPA-SC, family planning, implementation research, scale up, self-care

## Abstract

**Background:**

The introduction of self-administered injectable contraception presents an opportunity to address the unmet need for family planning. As ministries of health scale up self-administered injectable contraception, there is a scarcity of knowledge on the implementation practices and contextual conditions that help and hinder these efforts. The World Health Organization has launched the “enhancing self-administered family planning through embedded research project” (EASIER) to address this challenge.

**Objective:**

EASIER’s objectives are to: (1) assess the coverage of self-injectable contraception, and the readiness of health systems to integrate it into the contraceptive method mix; (2) document strategies used to introduce and scale up self-injectable contraception and understand practices that have led to success and challenges; (3) identify the contextual factors that affect the adoption and implementation of self-injectable contraception throughout health systems; (4) understand whether implementation addresses users’ preferences and needs; (5) strengthen collaboration between decision makers, researchers, and implementers; support and build capacity to use evidence.

**Methods:**

EASIER developed a global protocol that implementation research (IR) teams in Burkina Faso, Ghana, and Kenya adapted into country-level embedded IR projects. In all countries (1) at the national level, IR teams evaluate the policy environment for scaling up by conducting a desk review and in-depth interviews; (2) at the local level, IR teams implement quantitative questionnaires on structural and organizational readiness to integrate self-injection into the method mix; (3) in “case study” localities, IR teams conduct in-depth interviews and focus group discussions with implementers, method users, and community members; and (4) IR teams use participatory action research to elicit stakeholder participation and translate findings into programmatic decisions.

**Results:**

EASIER has been launched in all 3 countries. Preliminary findings are available from Burkina Faso and Kenya. In Burkina Faso, IR teams identified the need to strengthen health worker training approaches to ensure that family planning providers at primary health care facilities are adequately oriented to depo-medroxyprogesterone acetate subcutaneous (DMPA-SC) and self-injection and capacitated to initiate women to the method. In addition, they report the need for service communication strategies that reach potential users of the method with knowledge about self-injection and how to initiate the practice. In Kenya, the findings illuminate the need for practice guidelines that county health teams can use to coordinate the rollout of self-administered DMPA-SC. In addition, Kenya’s findings underscore the importance of addressing logistical bottlenecks to help avoid stock-outs.

**Conclusions:**

EASIER presents a strategy to embed IR in contraceptive method introduction and scale-up, address local knowledge needs, devise ways to maximize the impact of new technologies in health systems, and build capacity for using evidence in programmatic decisions. Adaptation and implementation of country-level IR studies will advance the use of IR to strengthen family planning programs.

**Trial Registration:**

Australia New Zealand Clinical Trials Registry ACTRN12622001228774; https://www.anzctr.org.au/Trial/Registration/TrialReview.aspx?id=384534&isReview=true

**International Registered Report Identifier (IRRID):**

DERR1-10.2196/44222

## Introduction

### Overview

In recent years, self-care interventions have received attention in the global health community because of their unique potential to alleviate constraints on individuals’ access to services and the pressure of increased service use on health systems [1]. Self-care encompasses manifold strategies that emphasize the needs and abilities of individuals, families, and communities to promote health, prevent and control disease, facilitate self-medication, provide care to dependent persons, rationalize the use of formal health care services, and facilitate rehabilitation [2,3]. In doing so, self-care can empower vulnerable communities to go beyond conventional health system responses and meet their needs despite health care worker shortages, humanitarian conflict, disease outbreaks, geographic barriers, and rising out-of-pocket health expenditures [4].

Unintended pregnancy, resulting from the unmet need for contraception, threatens the lives and well-being of girls and women globally. An estimated 214 million women in low- and middle-income countries (LMICs) have an unmet need for a modern method of contraception, and over half of these women live in sub-Saharan Africa and South Asia. According to the Guttmacher Institute, if all such women’s demand for contraception were satisfied, there would be a 75% decline in unintended pregnancies and 76,000 fewer maternal deaths per year [5]. Since 2013, evidence on the potential of self-care approaches in family planning (FP) has emerged. It builds on the accumulation of lessons from studies that demonstrated the safety, effectiveness, and acceptability of a woman-centered intervention for increasing access to the most popular contraceptive method in sub-Saharan Africa, depo-medroxyprogesterone acetate (DMPA) [6]. DMPA subcutaneous (DMPA-SC) can be administered by trained persons, including users themselves [7]. In 2019, the World Health Organization (WHO) issued global guidelines for sexual and reproductive health self-care interventions, which encouraged member states to introduce self-injectable DMPA into the method mix and scale up access to the method throughout health systems [1].

Ministries of health (MOHs) in LMIC and FP stakeholders require knowledge on ways to harness the potential of self-care to improve the effective coverage, acceptability, and use of national FP programs and strategies to maximize the impact of self-injection for women. Guidance on scaling up access to new contraceptive methods has emphasized the need to embed research in introduction processes and support implementers to learn, adapt, and use new knowledge to improve how health systems incorporate new technologies and reach users with them as part of a broader method mix [8-10]. Facilitating these opportunities in health systems can help ensure that FP managers and practitioners have the knowledge, tools, and support necessary to promote self-care, integrate self-injection into the method mix, and assure its availability to populations consistently and in a manner that meets clients’ needs. Implementation research (IR)—the systematic, scientific approach to determine how to get evidence-based interventions (EBI) to people who need it with greater speed, fidelity, efficiency, quality, sustainability, and relevant coverage—can help address this gap [11]. IR emphasizes collaboration between policy makers, decision makers, implementation teams, and local scientists who cocreate studies that are embedded in the reality of implementing organizations and address challenges to the optimal reach, delivery, and sustainment of EBI. Additional defining characteristics of IR are responsiveness to local demand for information and the positioning of research within planning and decision frameworks that use findings to improve services and systems [12].

In 2020, the WHO Department of Sexual and Reproductive Health and Research started the “enhancing self-administered family planning through embedded research” project (EASIER). EASIER is a global project which draws upon IR as a tool to help MOHs and their partners identify and evaluate programming strategies to best integrate self-injectable contraception into the method mix and support the spread of the method throughout health systems. The EASIER concept originated at the WHO based on requests from member states for support with monitoring and evaluating method introduction and scale-up. In 2021, EASIER launched an IR collaboration with partners in Burkina Faso and Kenya, and in 2022, it did so in Ghana. Although the EASIER approach emphasizes contextualization of IR studies to local conditions, across its applications, EASIER also emphasizes (1) partnerships between MOHs, local implementation teams, and local research institutions focused on identifying implementation challenges and positioning research within program processes; (2) building capacity for data use and knowledge translation so that IR partners cocreate solutions to implementation challenges based on evidence; and (3) the dissemination of lessons learned within and across countries to help improve self-injection program implementation at scale.

To launch EASIER, the WHO developed a global protocol. The global protocol provides IR teams in different countries with common research questions, methodological guidance, and data collection instruments that they can adapt and use to facilitate systematic learning about introducing self-administered DMPA-SC and scaling up. The protocol adopts a focus on clarifying the programmatic strategies countries have used, the contextual factors that helped or hindered implementation, and the implementation challenges and outcomes that have arisen as a result. In addition, it includes guidance on how IR teams can parlay evidence generation into the design of strategies to address implementation challenges. To date, the protocol has been adapted by IR teams in Burkina Faso, Ghana, and Kenya to develop site-specific IR projects. The purpose of this paper is to describe the global protocol and, in doing so, promote opportunities for global FP stakeholders to embed IR into the rollout of new contraceptive methods, or other EBI, in health systems. In addition, the paper provides readers with access to EASIER’s data collection instruments.

### EASIER Global Protocol: Objectives, Theoretical Influences, and Teams

The global protocol lays out the following objectives for country-level IR teams to adapt in site-specific IR protocols:

1. Understand the coverage of self-administered DMPA-SC and the readiness of national and local health systems to integrate it into the contraceptive method mix.
2. Document strategies used to integrate self-administered DMPA-SC into the method mix, identify practices that have led to success and challenges, and obtain insight on why these did or did not work
3. Identify the contextual factors that affect the adoption and implementation of self-administered DMPA-SC throughout the health system, including service provision and user settings where the method is practiced.
4. Understand user perspectives on their demand for the method and the degree to which their preferences and needs are addressed through implementation.
5. Strengthen collaboration between decision makers, researchers, and implementers; support and build capacity to use evidence to plan and improve implementation.

**Figure 1:**
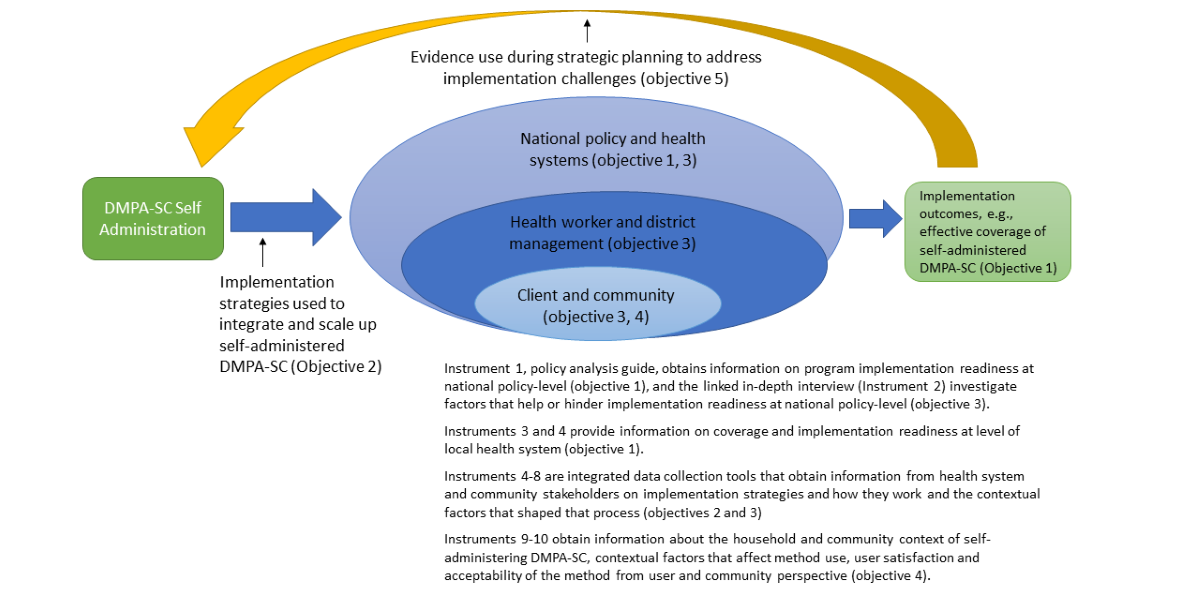
EASIER (enhancing self-administered family planning through embedded research project) global protocol objectives and instruments. DMPA-SC: depo-medroxyprogesterone acetate subcutaneous.

To develop these objectives, the WHO synthesized elements from multiple theories and frameworks, notably Social Ecological Theory and the Consolidated Framework for Implementation Research (CFIR) [13,14]. The former, which has been applied in public health to understand health behavior change, purports that health behaviors take shape and change as the product of numerous factors that emanate from multiple levels of context and affect individuals independently and synergistically. To adapt this perspective to the study of health care systems, we blend it with the CFIR, a determinants framework that guides the study of evidence-based interventions in complex systems and why they succeed or fail. Combining these frameworks was appropriate for the EASIER global protocol, given that its goal is to understand how implementation outcomes related to introducing DMPA-SC self-administration in systems were shaped as a product of strategic implementation by actors from multiple levels of health systems (eg, community, facility, district management, and national policy) and its interaction with contextual factors which also play out at multiple levels of context. The global protocol also reflects insights from the Promoting Action on Research Implementation in Health Services framework to develop guidance on research use approaches related to objective 5 [15] (Figure 1).

**Figure 2:**
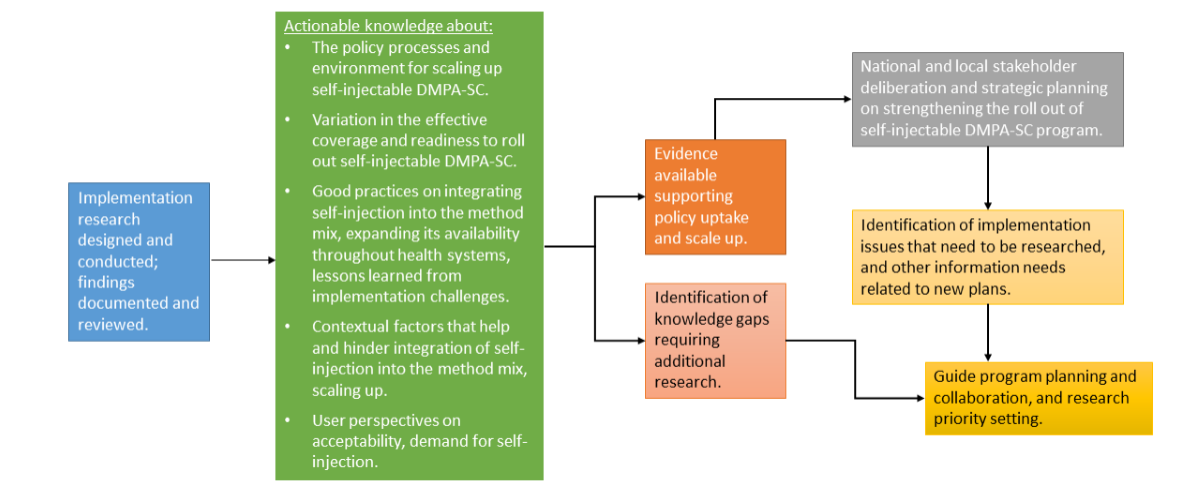
Role of implementation research in EASIER (enhancing self-administered family planning through embedded research project). DMPA-SC: depo-medroxyprogesterone acetate subcutaneous.

The EASIER global protocol objectives reflect the need to integrate knowledge on the performance of EBI in health systems from multiple domains of program implementation, which, in the case of self-administered DMPA-SC, includes users themselves and the communities in which they are nested, as well as the need to support structures for collaborative learning, adaptation, and evidence use. Figure 2 illustrates how IR will guide efforts to integrate and support the spread of self-injectable contraception in health systems.

The research methods and procedures described in this paper are adapted into country-specific protocols in Burkina Faso, Ghana, and Kenya. To catalyze this process, staff from WHO global headquarters and country offices engage MOHs in selecting decision makers from national and local levels of health systems to serve as principal investigators (PI) of country-level IR. The role of the PI is to position the IR within policy and program implementation cycles, ensuring that research is integrated with processes used by ministries, local health management teams, and implementation partners to introduce self-injection into the method mix and scale it up. PIs have a prominent role throughout the research process, establishing partnerships with local research institutions with whom they work to identify specific implementation issues to address, select and adapt methodologies, oversee data collection, interpret results, and stimulate the use of findings in programmatic decisions. In other words, decision makers in the EASIER project are both “knowledge producers” and “knowledge consumers.” In all countries, WHO country offices play a central role in helping to facilitate interactions between members of collaborative research partnerships and ensure that technical assistance is available to them when needed. See Table S1 in Multimedia Appendix 1, which describes the technical assistance that the WHO provides to in-country IR teams.

## Methods

### Study Environments and Design

The sections that follow describe the study design and data collection methods and procedures that are described in the EASIER global protocol. It calls for data collection to be carried out: (1) at the national level, where IR teams will evaluate the policy environment, assets, constraints, and processes for introducing and supporting the scale-up of DMPA-SC for self-administration; (2) at the local government level (eg, district, county, and provincial) in which MOHs have introduced DMPA-SC self-administration, where IR teams will assess implementation strength, service availability, and readiness to integrate the new method; and (3) in up to 6 “case study” districts (or counties and provinces) where IR teams will conduct in-depth qualitative research. The global protocol advises local IR teams on using maximum variation sampling techniques to select a geographically representative sample of districts for the qualitative case studies. Within each level of organization (national, case study county, and community), the research will sample individuals to participate in the study. The research will adopt a mixed methods design, including (1) qualitative methods at the national level and within case study countries to address objectives 1-4, that is, desk reviews of policy-related materials, in-depth interviews (IDIs) and focus group discussions (FGDs); (2) quantitative methods at the local government level in areas where governments’ have introduced self-injection to appraise implementation strength and readiness to integrate self-injection into the FP method mix (also objective 1); and (3) participatory action research (PAR) at the national level and within the case study counties to enable evidence use in programmatic decisions (objective 5).

### Sampling and Procedures

#### Objective 1

Lessons learned from previous WHO guideline introduction activities in countries revealed that the absence of policy implementation guidance represents a chief barrier to the incorporation of WHO FP guidelines in member states’ policy processes [16]. Other studies of evidence use in policy environments illuminate how this challenge plays out at the upstream stages of policy making and further downstream, where local health teams (eg, district health management teams) implement health policy [17]. To address these challenges, the EASIER global protocol describes methods IR teams can use to (1) understand the national-level policy environment for scaling up self-administered DMPA-SC at scale in countries and (2) gauge self-injection policy implementation at subnational (eg, district- and provincial-level) levels within countries. Regarding the policy environment, the study will use a policy analysis guide and IDI guide (see Instruments 1 and 2 in Multimedia Appendix 2). IR teams will use the former to review policies, guidelines, and other strategic documents relevant to the national policy and programmatic changes that were required to introduce self-injection into health systems. They will conduct IDI to understand more about the processes, arrangements, capacities, and resource considerations in relation to policy implementation generally and with respect to DMPA-SC self-administration, as well as participants understandings of the factors that have helped and hindered national-level efforts to introduce self-injection into the method mix. Researchers will first conduct the desk review and use the findings from it to customize lines of inquiry for the subsequent IDI. Purposive and snowball sampling approaches will be used to select the stakeholders for IDI. Before the interviews, stakeholders will be asked to supply IR teams with any documents relevant to the desk review that the latter were unable to obtain independently or through their professional networks. IDI participants will include MOH officials, FP experts, donor representatives, representatives from private sector nongovernmental implementing partners, private for-profit and private not-for-profit sector organizations, and health care professional organizations. For the IDIs, the sample size may vary across countries; however, the EASIER global protocol suggests that IR teams select 3 respondents from each of the 8 categories listed above (n=24). To be selected for IDIs, key informants will have to be involved with the DMPA-SC program and belong to any of the groups identified above. IR teams, in consultation with the MOH and WHO county offices, will draw a list of potential key informants from these groups. IDI informants will include those with knowledge of the reviewed documents and be available to clarify gaps in aspects of policies and pertinent issues that were not clear in the document review.

To help IR teams measure and compare across districts the degree to which local authorities have adopted and delivered self-administered DMPA-SC and the status of implementation, the EASIER global protocol has developed 2 instruments for local adaptation and use. The first is a coverage and structural readiness assessment (see Instrument 3 in Multimedia Appendix 3). In the districts (or counties) in which the government has authorized the rollout of self-administered DMPA-SC, IR teams will request leaders of local health management teams to complete this assessment, which asks for information on the availability of equipment, supplies, commodities, and infrastructure, human resources, health management information systems, supervision capacity, task shifting, community outreach, planning processes and support, financial resources, orientation to DMPA-SC guidelines, training capacity, etc. The second instrument is an organizational readiness for implementing change assessment (see Instrument 4 in Multimedia Appendix 4). For this, IR teams will enroll an additional 20 participants per implementing district (or county) to complete a questionnaire that elicits participants’ perceptions of DMPA-SC self-administration, organizational culture, the existing evidence base on the EBI, the capacity of their organization to adopt self-injection as a part of the method mix and implement it, the commitment of the organization to implement it, leadership, management, and teamwork issues. Participants in this questionnaire may include district directors of health services, district reproductive health focal persons, officers responsible for supply chain, health information systems, and community health services, and private sector (ie, nongovernmental organization and private pharmacy) representatives involved in DMPA-SC.

#### Objectives 2 and 3

Despite their differences, all programs to introduce self-injection in primary health care settings have employed good practices and experienced challenges from which valuable learning can arise. Further, they are all subject to contextual influences that have helped or hindered programs’ ability to effectively integrate and expand coverage of the new method. Understanding how these factors affect implementation effectiveness is needed to guide programmatic decisions to strengthen the scale-up of DMPA-SC self-administration in health systems. To address this information need, the EASIER global protocol provides IDI and FGD guides for IR teams to include in their qualitative case studies. These are integrated data collection tools that elicit data on the innovation characteristics and implementation strategies, whether they are effective or not, and why (objective 2), and explore how contextual factors at the district and community levels influence the DMPA-SC self-administration interventions (objective 3; see Instruments 5-8 in Multimedia Appendices 5-8). Participant selection will ensure representation of stakeholders from local health management teams, health facilities, other sectors, and communities whose insights will generate strategically relevant information on best practices, barriers and facilitators to effective implementation, and needs for adapting and enhancing interventions used to introduce and scale up the method. IR teams will purposively select district-level IDI participants, including district medical officers, reproductive health focal persons, district-level staff responsible for method procurement and supply chain logistics, health information, and community health services; representatives of nongovernmental organization involved with DMPA-SC; private sector clinicians and pharmacy workers that deliver the method; public sector health workers; and facility in-charges. At the community level, within each case study, the following groups of people will be purposively selected for the IDIs: community leaders, community health volunteers, influential men and women from communities where self-injection has been introduced, and persons that volunteer for health activities in the community. To obtain insights on collective attitudes and beliefs about themes that emerged in IDI, IR teams will enroll groups of people from each category of participants that had participated in objective 2 and 3 IDIs at the county and community levels in FGDs. These will include public health care workers, staff from private sector venues, community leaders, and members. Among health care professionals, FGD will be segmented according to sector (public and private) and rank. At the community level, FGD will be segmented by sex. In each case study locale, IR teams are advised to conduct 20 IDIs and 3 FGDs. Each FGD should include no more than 8 participants.

#### Objective 4

Strategies to integrate new interventions into health systems are prone to failure if they are not optimized to address user attitudes and characteristics. Similarly, interventions that are not attuned to the realities of the wider social and cultural environment may not reach target populations effectively or change behaviors. Accordingly, the EASIER global protocol incorporates “demand-side” research. This component of the study aims at understanding the household, community, and sociocultural context of introducing self-injectable contraception, how these factors affect demand for the new method, and ways in which implementation can be optimized to address women’s preferences and perceived needs. To obtain information from self-injection clients, the EASIER global protocol recommends that IR teams enroll 6 women per case study district in IDI that takes place 3 months after they initiated DMPA-SC self-administration (see Instrument 9 in Multimedia Appendix 9). The selection criteria for recruitment in these IDIs are that, upon their first interview, women were doing self-injection for the first time. IR teams should segment enrollment in these IDIs by age to ascertain a sample of an equal number of women younger than 25 years, 25-34 years, and 35 years or older, and complete interviews in private settings near participants’ homes or the closest health care facility. Interviewers will ask participants to discuss their demand for contraception and the factors that condition it, the acceptability and accessibility of DMPA-SC self-administration services, their perceived needs from a FP program, whether their expectations and needs have been met, their satisfaction and experience with the method, and reasons for continuation, or discontinuation or method switching. The success of implementation also depends on the extent to which programs are attuned to the norms and realities of the communities targeted by interventions. Accordingly, the EASIER global protocol recommends IR teams to carry out FGD in case study locations with community members, such as opinion leaders, DMPA-SC users, spouses of DMPA-SC users, and religious leaders (see Instrument 10 in Multimedia Appendix 10). FGD will elicit from community members their perception regarding the compatibility between interventions used to introduce DMPA-SC self-administration and communities’ shared beliefs, practices, and attitudes with respect to, for example, gender, religion, social relations, sexual and reproductive health, and the need for FP services. In addition, FGD will ask participants to reflect on the ways in which implementation can be adapted according to such factors, including influential social and communication networks that may influence the nature of demand for self-injection and FP services in general. The global protocol recommends that IR teams enroll 8 respondents per FGD in 3 FGDs per case study county.

#### Objective 5

The global protocol calls on IR teams to conduct PAR with study participants following the completion of Objectives 1-4 research and the analysis of preliminary findings. The purpose of PAR in EASIER is to engage participants in using evidence and translating findings into programmatic decisions toward improving the coverage, implementation, and sustainability of self-administered DMPA-SC services. The global protocol suggests that IR teams convene PAR at the national and case study levels and limit participation to individuals that were enrolled in IDI. IR teams are advised to conduct 2 PAR sessions at the national level: the first is a dissemination meeting of national stakeholders, which will involve those who participated in the national-level formative research (n=24) and up to 26 other relevant stakeholders from policy, programming, and donor circles (n=50); the second is a smaller meeting that will comprise of key national stakeholders (n=15) who will deliberate on findings and agree on implementation strategies to prioritize in the future. During PAR sessions at the national (session 1) and case study levels, IR teams will embed facilitators and rapporteurs in the large group of participants and break participants into small groups in which facilitators will guide discussions on the evidence, encouraging participants to brainstorm root causes of problems identified and understandings of why good practices were effective, rank and prioritize implementation issues that need to be addressed, discuss solutions, and recommend next steps for strengthening the introduction and scale up of DMPA-SC self-administration. Crucially, the case study-level PAR sessions will include community members and method users that participated in Objective 4 research. Their participation will be crucial to ensure that next steps reflect the perspectives of current and potential clients and the need for person-centered implementation strategies. Rapporteurs will document these discussions, recording data in a manner that does not include identifiable or personal information about PAR participants. Data collected during PAR will comprise of rapporteur reports and notes from small group activities and plenary discussions. The same data collection will be carried out in the first of the national- and case-study-level PARs. For the second national-level meeting, the research team will take notes and disseminate a meeting report.

### Analytical Approaches

#### Objective 1

The EASIER global protocol advises IR teams on ways to triangulate qualitative analysis methodologies, including content analysis of documents analyzed from the desk review and framework analysis of IDI, to depict the policy-making and implementation characteristics, capacities, and processes in relation to the public sector rollout of DMPA-SC self-administration at the national level [18,19]. Data from the structural and organizational readiness questionnaires will be analyzed through univariate and bivariate statistical procedures. IR teams will generate descriptive analyses to capture variation across districts in terms of levels of adoption of the new method, structural readiness, and organizational readiness to scale it up. They will draw on these data to formulate dependent variables and carry out exploratory analysis to identify factors that are associated with that outcome. This will include correlation analyses between independent variables identified from these data sets to assess potential collinearity between measures. This will inform the basis for potential multivariate analysis that brings in adjustment terms and provide a richer understanding of factors in our data that are associated with readiness for implementation and implementation strength.

#### Objectives 2-4

The global protocol outlines procedures for analyzing the qualitative data collected in each case study location. These data will be analyzed independently to obtain a rich picture within each case study, respectively, of the implementation process–related and contextual factors that influence the adoption and delivery of DMPA-SC self-administration and the outcomes that emerge as a product of context-process interactions. This analysis will reflect the theoretical influence of contextual analysis, a concept from “realistic evaluation” that is based on the principle of “generative causation” [20]. Contextual analysis holds that what works and for whom is contingent upon the circumstances in which initiatives are implemented. The global protocol suggests that IR teams blend framework analysis and steps adapted from grounded theory analysis to, first, identify segments of the qualitative data that reflect the context of self-injection implementation, implementation processes and characteristics, and implementation outcomes [18,21]. IR teams should then draw on methods such as “constant comparison” to compare data from alternative transcripts within each case study to generate preliminary causal pathway models (CPM) that connect implementation strategies, preconditions, implementation mechanisms, and processes with proximal and, if possible, distal outcomes [22,23]. Based on this, IR teams will generate a codebook with codes to represent each of the components of the CPM for each case study and use these to analyze the qualitative data. IR teams will formulate “context-mechanism-outcome” (CMO) configurations based on the coded data for each case study [20]. They will compare each CMO configuration against each preliminary CPM to assess the strength of the explanatory framework they have posited for each case study and the need for modifications and refinements. Depending on their conclusions, they may revert to the data analysis to carry out additional phases of coding to integrate concepts included in the initial codebooks for refined analyses (ie, axial coding), and, after this, research team members will review reports from the second rounds of coding, formulate CMO configurations, and re-examine their CPM [21]. This cycle will continue until IR teams reach consensus on their satisfaction with CPMs that are finalized based on CMO analyses. With this, IR teams will generate explanations for how contextual factors and implementation mechanisms shape the outcomes of self-injection implementation in case study locations. The research team will compare the CMO analyses and final CPM of each case study to obtain a nuanced understanding of the determinants, processes, and outcomes of DMPA-SC self-administration introduction in each country.

#### Objective 5

Each PAR meeting will be led by an experienced moderator and small group discussion facilitators. IR teams will present salient findings from the research and then divide the plenary group of each dissemination into small groups for discussion. Data collectors will be embedded in each discussion to take detailed notes and facilitate group discussions using flipcharts, markers, and other materials as necessary. The underlying purpose of PAR activities described above, such as reflection on root causes of problems or drivers of implementation success, is to engender among participants the capacity and impetus for “systems thinking” (ie, seeing wholes, perceiving structures that underlie dynamically complex systems, and identifying high leverage opportunities), and the elaboration of “mental models” (ie, reflection, inquiry, testing, and improving of assumptions about how things work within organizations) [24]. Using these tools, facilitators will guide small groups in discussion. During discussion, these groups will prioritize implementation issues to address in the future and suggest actions that should be taken to do so. Large group discussions will proceed, facilitated by the moderator, during which small groups will present and debate their priorities. This will continue until the large group achieves consensus on a limited set of future activities associated with the next stages of the DMPA-SC self-administration scale-up. A multistep ranking process will ensue whereby each participant ranks, individually and confidentially, each of the selected activities. The moderator and data collection team will review the activity rankings submitted by each participant and calculate for each activity the number of submissions that ranked it number 1, number 2, number 3, etc until all activities have been prioritized. The intention of this activity is to establish a shared vision for future scale-up within each case study and at the national level. This will continue until each activity is ranked in an action plan or discarded. Altogether, each PAR session should take 1 full working day.

### Ethics Approval

This research protocol has been reviewed by the research project review panel (RP2), an external scientific review body that is assembled by the Department of Sexual and Reproductive Health and Research of WHO to review the department’s research protocols, as well as the WHO Ethical Review Committee (protocol number WHO ERC A66003) and the adapted country protocols registered in the respective country’s local institutional review boards.

## Results

As of October 2020, EASIER started data collection in Kenya and Burkina Faso and started the process of protocol adaptation in Ghana. In preliminary analysis of the data from Kenya, a key emerging theme is the challenges that arise at the local management and service delivery levels owing to the absence of practice guidelines. Although policies and training curricula are in place, implementation teams report that they lack a clear strategy on how to operationalize this policy decision. To address this, teams ask that practice guidelines provide operational detail on how county health teams should roll out the new method. Specifically, participants ask for guidance on how county health teams should adapt training, counseling, routine supervision, quality assurance, health information, and community outreach procedures to optimize the introduction of the new method and best leverage existing community-based and private sector delivery channels. A second key theme from Kenya concerns lapses in the availability of DMPA-SC commodities, which arise from bottlenecks in the procurement and distribution of the method at the national and local levels. Preliminary findings have been shared with the MOH and remedial actions are underway to address challenges.

In Burkina Faso, preliminary findings suggest that the effort to scale up self-administered DMPA-SC has achieved a relatively large scale in the country. However, health workers based at the lowest levels of care were not adequately trained to initiate women’s self-injection. Although investment has been made in expanding coverage of the method, the voices of stakeholders indicate that the MOH and implementation partners should consider reforming scale-up processes to ensure that staff at primary health care facilities, which are usually closest to communities, are reached with the training they require to become able to initiate and support clients on self-injection. A second emergent theme that has arisen from preliminary analysis of the Burkina Faso IR data relates to women’s awareness of the opportunity to practice self-injection. Despite efforts to make the method available, most women in the sample indicated that they had not been informed of the opportunity to self-inject DMPA-SC. The findings further imply that devising strategies to leverage community-based structures and other service communication channels to spread understanding of self-administered DMPA-SC and its availability will be crucial to strengthening the scale-up of the method. Preliminary evidence from the IR project in Ghana will become available in January 2023.

## Discussion

### Overview

We expect that both the quantitative and qualitative pieces of these studies will be robust. Specifically, we anticipate that the former will generate important information on the variation of coverage and implementation strength of efforts to expand the method mix through self-administration of DMPA-SC and decisions on where future interventions are required in order to address disparities and gaps. In addition, we expect that qualitative data will inform the design of implementation strategies that FP programs in the 3 countries will use to enhance method introduction and scale-up processes. By engaging stakeholders from multiple levels of the countries’ health systems in PAR, we expect subsequent steps that aim at strengthening and accelerating scale-up efforts to be led by policy makers and decision makers and integrated into the implementation processes and cycles of local health care delivery teams. To facilitate this process, EASIER IR teams in each country receive support from the WHO to work with MOH counterparts and, as a group, reflect on Objectives 1-5 research findings to refine an implementation strategy that addresses priorities and challenges that were identified during PAR sessions. Based on this, IR partners in each country will develop an IR protocol to evaluate that strategy in a subsequent phase of the EASIER project. During this process, WHO will provide technical assistance to IR partners in each country, orienting them to evidence-based strategies that have been proven to address the specific issues that IR partners address in their implementation strategies, including the use of technology to enhance future implementation efforts (eg, training videos, user-clinic apps, SMS message reminder systems, etc). In addition, the WHO will support IR partners in adapting their chosen evidence-based approaches to their context and incorporating them into their plans.

### Strengths and Limitations

A strength of EASIER is that it leverages a variety of implementation science frameworks and approaches to understand outcomes that have emerged from early efforts to scale up self-administered DMPA-SC in countries as well as the generative process that explains differences in these outcomes across diverse geographies within countries. The use of a global protocol will help ensure that findings across countries are comparable and that collectively they can help advance the global evidence base on the barriers, facilitators, and good practices associated with implementation success and challenges with respect to the scale-up of DMPA-SC self-administration. Further, the WHO approach to using multidisciplinary teams that place decision makers and implementers at the helm of the IR and its use of PAR integrate IR and FP program processes and create opportunities for evidence use as part of scaling up. The EASIER protocol has limitations as well. The use of a global protocol implies a rigorous adaptation process at the country level to ensure that it addresses local knowledge needs and implementation challenges and maximally benefits local actors involved in scaling up. It is primarily focused on obtaining a thorough understanding of method introduction, scale-up progress, processes, and problems in order to pinpoint challenges and prescribe solutions based on evidence. It does not evaluate specific strategies aimed at accelerating scale-up or strengthening systems, even though EASIER’s findings can establish a strong foundation for doing so.

### Conclusions

The emergence of DMPA-SC for self-administration has the potential to transform access to the contraceptive method considered most popular in settings where better variety and access to FP are critical for achieving global health goals. Global organizations have recognized self-administered DMPA-SC as an EBI and recommended its inclusion in countries’ FP programs; however, countries lack contextually appropriate strategies for integrating it and supporting its introduction and scale-up in health systems. IR offers ways to address this gap by bringing into focus underlying implementation issues and challenges related to the introduction of new methods and explaining the implementation process–related and contextual factors that shape whether the introductory and scale-up efforts are effective. IR also supports knowledge translation frameworks through which stakeholders use evidence to make programmatic decisions on how to address challenges. The EASIER project demonstrates ways to embed IR within the health system and employ it to help guide scaling up access to self-injectable DMPA as part of contraceptive method choice. If successful, the protocol described in these pages will propagate additional cycles of learning, adaptation, implementation, and research, with the goal of supporting the spread of this promising contraceptive method based on local evidence in Burkina Faso, Ghana, Kenya, and additional countries that join the EASIER project. Altogether, this will create new and meaningful opportunities to advance the use of IR to strengthen FP programs.

## Appendix

Multimedia Appendix 1 Table 1: Technical Assistance offered by WHO (headquarters and country offices).

Multimedia Appendix 2 Instrument 1 and 2: Self-Administered DMPA-SC Policy Assessment Tool and National Level In-depth Interview Guide.

Multimedia Appendix 3 Self-Administered DMPA-SC Coverage and Structural Readiness Assessment.

Multimedia Appendix 4 Self-Administered DMPA-SC Program: Organizational Readiness for Change Assessment.

Multimedia Appendix 5 Subnational (eg, district-level) In-depth Interview Guide with local health system staff.

Multimedia Appendix 6 Subnational (eg, district-level) In-depth Interview Guide with members of communities and community organizations.

Multimedia Appendix 7 Subnational (eg, district-level) focus group discussion guide for local health system staff.

Multimedia Appendix 8 Subnational (eg, district-level) focus group discussion guide for community members and members of community organizations.

Multimedia Appendix 9 Self-Administered DMPA-SC Client In-depth Interview Guide.

Multimedia Appendix 10 Self-Administered DMPA-SC Community-level Focus Group Discussion Guide.

## Abbreviations

CFIR: Consolidated Framework for Implementation Research
CMO: context-mechanism-outcome
CPM: causal pathway model
DMPA: depo-medroxyprogesterone acetate
DMPA-SC: depo-medroxyprogesterone acetate subcutaneous
EASIER: enhancing self-administered family planning through embedded research project
EBI: evidence-based intervention
FGD: focus group discussion
FP: family planning
IDI: in-depth interview
IR: implementation research
LMIC: low- and middle-income countries
MOH: Ministry of Health
PAR: participatory action research
PI: principal investigator
RP2: research project review panel
WHO: World Health Organization

## Data Availability

The sharing of data used and analyzed during this study must adhere to the WHO data sharing policy [25]. Deidentified data used and analyzed during this study can be requested from the corresponding author. Data will be shared contingent on approval by the WHO Ethical Review Committee and local ethical review committees in the study countries.
