# Supplementary material for: Enhancing the Introduction and Scale Up of Self-Administered Injectable Contraception (DMPA-SC) in Health Systems (the EASIER Project): Protocol for Embedded Implementation Research
Source: JMIR Res Protoc. 2023 Aug 23;12:e44222. doi: 10.2196/44222 (PMC10483301; doi:10.2196/44222)
Supplement: Multimedia Appendix 1 [file resprot_v12i1e44222_app1.docx]

**Table S1: Technical Assistance offered by WHO (headquarters and country offices)**

| 1. Coordinate formation of in-country IR teams (e.g., engagement and obtaining buy-in from Ministries of Health, recruitment of Principal Investigators from Ministries of Health, facilitation of recruitment and selection of in-country research institution to implement the IR). |
| --- |
| 1. Orient in-country IR teams to the global protocol and instruments, and provide technical support to IR teams to help adapt global protocol into country specific IR studies, including development of country specific protocols. |
| 1. Convene routine meetings between IR partners and key stakeholders in countries to coordinate timelines, help ensure alignment between Ministry of Health program implementation cycles and research activities. |
| 1. Leverage dissemination channels to share research findings among stakeholders |
| 1. Provide technical support for knowledge translation, helping ensure that IR findings are utilized to develop programmatic decision-making alternatives and design implementation strategies to enhance method introduction and scale up processes. |
| 1. Harvest research findings from IR teams in different countries, convene fora for teams from different countries to exchange lessons learned and plan subsequent stages of the global IR project. |
