# Supplementary material for: Enhancing the Introduction and Scale Up of Self-Administered Injectable Contraception (DMPA-SC) in Health Systems (the EASIER Project): Protocol for Embedded Implementation Research
Source: JMIR Res Protoc. 2023 Aug 23;12:e44222. doi: 10.2196/44222 (PMC10483301; doi:10.2196/44222)
Supplement: Multimedia Appendix 2 [file resprot_v12i1e44222_app2.docx]

**Self-Administered DMPA-SC Policy Assessment Tool**

**Instructions:**

- This checklist is intended to guide the review of current policies, guidelines and other documents related to the national Self-Administered DMPA-SC program. Documents should be acquired from stakeholders knowledgeable of the program at the national policy level and level of local policy dissemination for whom policy-related documents are accessible. These persons will include central level Ministry of Health staff, regional, local health administrators, senior-level health providers and service supervisors.
- Review the documents and answer the questions below. For each “yes” answer (in section 1), in section 2, quantify the number of times the issue raised by each checklist item is discussed in the policy, and score the policy in terms of the clarity and comprehensiveness of that item. Provide salient examples, by citing traceable, specific sections of the document(s) reviewed.
- For each item in the checklist (Table 1) that receives a “no” answer, or a score of 2 or below in Table 2, formulate lines of questioning to later pose to stakeholders. The questions(s) should elicit reasons for the “no”, or low score, and stakeholders’ perceptions of the need to resolve this, how this might be achieved, and, if necessary, facilitate discussion on draft policy statements that will do so, based on evidence and on the country setting.
- Not all documents reviewed should address each of the issues raised in the checklist. Data collectors should use their judgment to determine this for the documents reviewed. When this arises, data collectors should mark “not applicable (NA)”.
- The final section leaves space for comments on difficulties experienced in completing the document analysis or any remarks that the data collector wishes to leave.

| Name of data collector:  ______________________________________________________________________  Date:  ______________________________________________________________________  Country:  ______________________________________________________________________  Name of Policy document and year of publication:  ______________________________________________________________________  Author of policy document (organization, individual):  _____________________________________________________________________  Purpose of policy document: ______________________________________________________________________  Level (national, local, international): ______________________________________________________________________  Means of acquiring the policy document:  ______________________________________________________________________ |
| --- |

1. **Document review checklist**

| Does the policy document about/related to the self-administered DMPA-SC program: | | Yes | No | NA |
| --- | --- | --- | --- | --- |
| 1 | Provide clinical guidelines for services. |  |  |  |
| 2 | Provide management guidelines for the program. |  |  |  |
| 3 | Articulate standards for services (i.e. standards of care, quality, safety). |  |  |  |
| 4 | Articulate a commitment to ensure access for all women. |  |  |  |
| 5 | Tell health managers and providers which services should be available. |  |  |  |
| 6 | Specify how often and under what conditions services are provided. |  |  |  |
| 7 | Articulate where the services are to be delivered. |  |  |  |
| 8 | Articulate what sectors deliver the service. |  |  |  |
| 9 | Articulate who is eligible for the services. |  |  |  |
| 10 | Identify which cadre of health personnel delivers DMPA-SC services. |  |  |  |
| 11 | Articulate expanded practices of non-traditional family planning service providers for the self-administered DMPA-SC program. |  |  |  |
| 13 | Require budgetary allocations for services within the overall health budget. |  |  |  |
| 14 | Articulate role/processes for supervision, quality assurance and improvement, or links to broader QA/QI processes. |  |  |  |
| 15 | Articulates training standards and competencies and modes of verification. |  |  |  |
| 16 | Articulates training modalities for developing necessary human resource capacity for the program. |  |  |  |
| 17 | Articulates measures for ensuring commodity security, requesting, procuring, and distribution of commodities for the program. |  |  |  |
| 18 | Discuss health information and reporting procedures. |  |  |  |
| 20 | Articulates the client-support requirements for the national program. |  |  |  |
| 21 | Articulates a role for multi-sectoral collaboration. |  |  |  |
| 22 | Discusses integration of DMPA-SC delivery with other health services. |  |  |  |
| 23 | Articulates free, full and informed choice to a wide range of contraceptive methods, including DMPA-SC. |  |  |  |
| 24 | Discusses client costs, social protections to promote affordability. |  |  |  |
| 25 | Aligns DMPA-SC program with overall national family planning strategies. |  |  |  |
| 26 | Aligns DMPA-SC program with other policies and policy frameworks. |  |  |  |
| 27 | Aligns DMPA-SC program with global frameworks (e.g. FP2020, SDG, etc.). |  |  |  |
| TOTAL [number ‘yes’, ‘no’, ‘NA’] | |  |  |  |

1. **Policy scoring table (for those marked yes above).**

| Does the policy document about/related to the self-administered DMPA-SC program: | | # times discussed in document | | Strength of policy articulation (1-5, 5=highest |
| --- | --- | --- | --- | --- |
| 1 | Provide clinical guidelines for services. |  |  | |
| Justification for score:  Page and paragraph numbers:  Salient quotations: | | | | |
| 2 | Provide management guidelines for the program. |  | |  |
| Justification for score:  Page and paragraph numbers:  Salient quotations: | | | | |
| 3 | Articulate standards for services (i.e. standards of care, quality, safety). |  | |  |
| Justification for score:  Page and paragraph numbers:  Salient quotations: | | | | |
| 4 | Articulate a commitment to ensure access for all women. |  | |  |
| Justification for score:  Page and paragraph numbers:  Salient quotations: | | | | |
| 5 | Tell health managers and providers which services should be available. |  | |  |
| Justification for score:  Page and paragraph numbers:  Salient quotations: | | | | |
| 6 | Specify how often and under what conditions services are provided. |  | |  |
| Justification for score:  Page and paragraph numbers:  Salient quotations: | | | | |
| 7 | Articulate where the services are to be delivered. |  | |  |
| Justification for score:  Page and paragraph numbers:  Salient quotations: | | | | |
| 8 | Articulate what sectors deliver the service. |  | |  |
| Justification for score:  Page and paragraph numbers:  Salient quotations: | | | | |
| 9 | Articulate who is eligible for the services. |  | |  |
| Justification for score:  Page and paragraph numbers:  Salient quotations: | | | | |
| 10 | Identify which cadre of health personnel delivers DMPA-SC services. |  | |  |
| Justification for score:  Page and paragraph numbers:  Salient quotations: | | | | |
| 11 | Articulate expanded practices of non-traditional family planning service providers for the self-administered DMPA-SC program. |  | |  |
| Justification for score:  Page and paragraph numbers:  Salient quotations: | | | | |
| 13 | Require budgetary allocations for services within the overall health budget. |  | |  |
| Justification for score:  Page and paragraph numbers:  Salient quotations: | | | | |
| 14 | Articulate role/processes for supervision, quality assurance and improvement, or links to broader QA/QI processes. |  | |  |
| Justification for score:  Page and paragraph numbers:  Salient quotations: | | | | |
| 15 | Articulates training standards and competencies and modes of verification. |  | |  |
| Justification for score:  Page and paragraph numbers:  Salient quotations: | | | | |
| 16 | Articulates training modalities for developing necessary human resource capacity for the program. |  | |  |
| Justification for score:  Page and paragraph numbers:  Salient quotations: | | | | |
| 17 | Articulates measures for ensuring commodity security, requesting, procuring, and distribution of commodities for the program. |  | |  |
| Justification for score:  Page and paragraph numbers:  Salient quotations: | | | | |
| 18 | Discuss health information and reporting procedures. |  | |  |
| Justification for score:  Page and paragraph numbers:  Salient quotations: | | | | |
| 20 | Articulates the client-support requirements for the national program. |  | |  |
| Justification for score:  Page and paragraph numbers:  Salient quotations: | | | | |
| 21 | Articulates a role for multi-sectoral collaboration. |  | |  |
| Justification for score:  Page and paragraph numbers:  Salient quotations: | | | | |
| 22 | Discusses integration of DMPA-SC delivery with other health services. |  | |  |
| Justification for score:  Page and paragraph numbers:  Salient quotations: | | | | |
| 23 | Articulates free, full and informed choice to a wide range of contraceptive methods, including DMPA-SC. |  | |  |
| Justification for score:  Page and paragraph numbers:  Salient quotations: | | | | |
| 24 | Discusses client costs, social protections to promote affordability. |  | |  |
| Justification for score:  Page and paragraph numbers:  Salient quotations: | | | | |
| 25 | Aligns DMPA-SC program with overall national family planning strategies. |  | |  |
| Justification for score:  Page and paragraph numbers:  Salient quotations: | | | | |
| 26 | Aligns DMPA-SC program with other policies and policy frameworks. |  | |  |
| Justification for score:  Page and paragraph numbers:  Salient quotations: | | | | |
| 27 | Aligns DMPA-SC program with global frameworks (e.g. FP2020, SDG, etc.). |  | |  |
| Justification for score:  Page and paragraph numbers:  Salient quotations: | | | | |

1. **Lines of questioning for identifying needs and approaches for policy improvement (for those marked no above).**

| Q# | Lines of questioning |
| --- | --- |
|  |  |
|  |  |
|  |  |
|  |  |
|  |  |

1. **Comments**

| Q# | Comments |
| --- | --- |
|  |  |
|  |  |
|  |  |
|  |  |
|  |  |

**National-level In-depth Interview: Contextual Influences on, and Programmatic Determinants of, the Outcomes of the national DMPA-SC Program.**

**Instructions:**

- This in-depth interview (IDI) is intended to obtain strategic information from key informants on (a) factors that influence the effectiveness, feasibility and sustainability of the DMPA-SC program, and (b) the features of the program that have shaped these outcomes of the program.
- Key informants should come from national-level organizations, or be influential independent stakeholders at the national-level, that can describe national- or global-level influences in response to objective (a) above. Key informants should draw upon their perspectives as national stakeholders to respond to objective (b).
- For both objectives of the IDI, participants should elucidate these factors and features, how they operate and why they affect the effectiveness, feasibility and sustainability of the DMPA-SC program. They should explain why and how they believe these factors and features will influence the scale up of the DMPA-SC program in their country.
- For questions concerning objective (a), key informants should describe how they think the DMPA-SC program needs to react in order to mitigate adverse contextual influences and leverage positive contextual influences.
- For questions concerning objective (b), key informants should describe if they think the salient features of the program that have shaped the outcomes of the DMPA-SC program should be continued during scale up, eliminated and why? They should describe if these features should be adapted as the DMPA-SC program goes to scale, and, if so, how and why.

| Name of data collector:  ______________________________________________________________________  Date:  ______________________________________________________________________  Country:  ______________________________________________________________________  Name of Key Informant:  ______________________________________________________________________  Job Title of Key Informant:  _____________________________________________________________________  Organization where Key Informant is employed: ______________________________________________________________________  Number of Years Key Informant has been in that job: ______________________________________________________________________  Role of Key Informant in the National DMPA-SC Program:  ______________________________________________________________________  Location of the interview:  ___________________________________________________________________________ |
| --- |

**Introduction:**

**INTERVIEWER SHOULD CONFIRM WITH THE KEY INFORMANT THAT S/HE HAS PERMISSION TO RECORD THE IDI ON A RECORDED DEVICE. IF RESPONDENT CONSENTS, THE INTERVIEWER CAN START RECORDING NOW. RECORD THE TIME AT WHICH THE INTERVIEW BEGINS.**

START TIME OF IDI: ______________________

***[READ ALOUD – INTERVIEWERS CAN PARAPHRASE]:*** *Thank you for taking the time to participate in this in-depth interview on the DMPA-SC program in [name of country]. As you are aware, the policies of [name of country] permit the use of DMPA-SC for self-administration. Women who desire the method, can be screened by a healthcare worker and, provided that they are eligible for the method, receive their first injection at the facility together with a re-supply kit of DMPA which they can inject into themselves, sub-cutaneously, at home after their initial injection has expired. We are interested in your perceptions of this national program, and the factors in the national, or global, environment, which you are familiar with in your role as [state the job title of the key informant] at [state the key informant’s place of work], that influence the program, its effectiveness, its feasibility and its sustainability. Secondly, we are interested your perception of the features of the national program of DMPA-SC that you believe are responsible for the outcomes of the program – the program’s effectiveness, feasibility and sustainability. Finally, reflecting on the factors and programmatic features you discuss, we would like your views on what the program needs to do in the future as it pursues scale up.*

**Interview:**

1. **CONTEXTUAL INFLUENCES ON THE NATIONAL DMPA-SC PROGRAM**
   1. *Please draw upon your knowledge and experience with this program in your country, and describe the design of the national program of self-administered DMPA-SC in [name of country]. In other words, based on your understanding, how is the DMPA-SC program supposed to operate in your country?*

[**NOTE TO INTERVIEWER:** IF NECESSARY, PROBE FOR SPECIFIC ASPECTS OF THE NATIONAL DESIGN OF THE PROGRAM:

- WHO PERFORMS THE SERVICE AND WHERE
- CLINICAL STANDARDS AND GUIDELINES
- WORKFORCE READINESS AND AVAILABILITY
- TRAINING AND TASK SHARING
- LOGISTICS, SUPPLY CHAIN AND COMMODITY SECURITY
- ROLE OF THE COMMUNITY
- HOW THE PROGRAM IS FINANCED
- COST RECOVERY/USER FEES
- MONITORING, INDICATORS AND HEALTH INFORMATION SYSTEMS
- INSURANCE, FEE EXEMPTIONS AND SOCIAL PROTECTIONS
- NATIONAL PROGRAM MANAGEMENT AND COORDINATION STRUCTURES
- NATIONAL POLICIES RELATED TO THE NATIONAL PROGRAM
- DONORS, IMPLEMENTING PARTNERS AND THEIR ROLES
- OTHER SECTORS INVOLVED IN THE NATIONAL PROGRAM AND THEIR ROLES

THE KEY INFORMANT DOES **NOT** HAVE TO PROVIDE INFORMATION ON ALL ASPECTS OF THE PROGRAM; HOWEVER PROBE REPEATEDLY, BUT SENSITIVELY, TO ELICIT AS MUCH INFORMATION AS REALISTICALLY POSSIBLE.]

- 1. *As I mentioned in the introduction, we would like to understand your perceptions of factors in the national, and possibly global, environment that influence the program – positively and negatively. Reflecting on the elements of the design of the national DMPA-SC program, let us take a moment to brainstorm the factors that influence if these design elements are successful.*

[NOTE TO INTERVIEWER: IF NECESSARY, PROBE FOR SPECIFIC TYPES OF FACTORS AND PROMPT THE KEY INFORMANT TO DESCRIBE WHAT THESE ARE IN RELATION TO THE INDIVIDUAL FACTORS MENTIONED IN RESPONSE TO Q1.1. SPECIFIC TYPES OF FACTORS ARE:

- LEADERSHIP (E.G. CHANGES IN LEADERSHIP, OPINIONS, POSITIONS, AND ATTITUDES OF CURRENT LEADERS, E.G. POLITICAL, RELIGIOUS, ETC).
- DONOR ENVIRONMENT AND TRENDS
- REVISIONS TO POLICY, UPCOMING POLICY PLANNING ACTIVITIES.
- CLINICAL UPDATES AND TRENDS.
- HEALTH SECTOR BUDGETING AND FINANCING TRENDS
- NATIONAL PROCUREMENT AND DISTRIBUTION PROCESS (CHANGES IN PROCESSES)
- ECONOMIC CONDITIONS IN THE COUNTRY
- INFLUENCE OF NATIONAL PROFESSIONAL ASSOCIATIONS
- OTHER EMERGING PUBLIC HEALTH PRIORITIES
- NATIONAL WORKFORCE ISSUES AND CHALLENGES

THIS QUESTION ASKS FOR BRAINSTORMING OF FACTORS IN RELATION TO DESIGN ELEMENTS MENTIONED IN Q1.1, NOT EXHAUSTIVE EXPLANATION OF HOW THE FACTORS INFLUENCE THE PROGRAM. THE KEY INFORMANT DOES **NOT** HAVE TO PROVIDE INFORMATION ON ALL POTENTIAL CONTEXTUAL FACTORS. MAKE SURE THAT YOU OR THE IDI NOTETAKER DOCUMENTS THE FACTORS MENTIONED AND THE CORRESPONDING PROGRAM DESIGN ELEMENT.]

- 1. *Just to confirm, you have identified the following contextual factors affecting the following elements of the national program of DMPA-SC.*

[**NOTE TO INTERVIEWER:** SUMMARIZE ALOUD, SUCCINCTLY, THE CONTEXTUAL FACTORS MENTIONED IN Q1.2 AND THE CORRESPONDING PROGRAM DESIGN ELEMENTS MENTIONED IN Q1.1]

*Can you rank in the order of most influential to least influential the three most influential contextual factors you discussed moments ago? Explain your justification for ranking these as you do.*

- 1. *Reflecting on the most influential contextual factor, can you elaborate on how it affects the outcomes of the program that you have observed to date?*

[**NOTE TO INTERVIEWER:** PROBE SPECIFICALLY FOR KEY INFORMANTS PERCEPTIONS OF THE INFLUENCE OF FACTORS ON OUTCOMES ON EFFECTIVENESS, FEASIBILITY TO DELIVER THE INTERVENTION THROUGH THE NATIONAL PUBLIC HEALTH SYSTEM, SUSTAINABILITY OF THE NATIONAL PROGRAM]

- 1. *Reflecting on the second most influential contextual factor, can you elaborate on how it affects the outcomes of the program that you have observed to date?*

[**NOTE TO INTERVIEWER:** PROBE SPECIFICALLY FOR KEY INFORMANTS PERCEPTIONS OF THE INFLUENCE OF FACTORS ON OUTCOMES ON EFFECTIVENESS, FEASIBILITY TO DELIVER THE INTERVENTION THROUGH THE NATIONAL PUBLIC HEALTH SYSTEM, SUSTAINABILITY OF THE NATIONAL PROGRAM]

- 1. *Reflecting on the third most influential contextual factor, can you elaborate on how it affects the outcomes of the program that you have observed to date?*

[**NOTE TO INTERVIEWER:** PROBE SPECIFICALLY FOR KEY INFORMANTS PERCEPTIONS OF THE INFLUENCE OF FACTORS ON OUTCOMES ON EFFECTIVENESS, FEASIBILITY TO DELIVER THE INTERVENTION THROUGH THE NATIONAL PUBLIC HEALTH SYSTEM, SUSTAINABILITY OF THE NATIONAL PROGRAM]

- 1. *Reflecting on the most influential contextual factor, can you elaborate on how you think it will affect the prospect of scaling up the national DMPA-SC program? What do you believe that the program should do in response to the influence of [state the most influential contextual factor]?*

[**NOTE TO INTERVIEWER:** FOR THE LATER COMPONENT OF 1.7, PROBE FOR WAYS IN WHICH THE DESIGN ELEMENTS OF THE PROGRAM MAY NEED TO CHANGE, LEADERSHIP DECISIONS THAT SHOULD BE MADE, MANAGEMENT STRATEGIES FOR MITIGATING THE ADVERSE EFFECTS AND/OR LEVERAGING THE POSITIVE EFFECT OF FACTORS].

- 1. *Reflecting on the second most influential contextual factor, can you elaborate on how you think it will affect the prospect of scaling up the national DMPA-SC program? What do you believe that the program should do in response to the influence of [state the most influential contextual factor]?*

[**NOTE TO INTERVIEWER:** FOR THE LATER COMPONENT OF 1.8, PROBE FOR WAYS IN WHICH THE DESIGN ELEMENTS OF THE PROGRAM MAY NEED TO CHANGE, LEADERSHIP DECISIONS THAT SHOULD BE MADE, MANAGEMENT STRATEGIES FOR MITIGATING THE ADVERSE EFFECTS AND/OR LEVERAGING THE POSITIVE EFFECT OF FACTORS].

- 1. *Reflecting on the third most influential contextual factor, can you elaborate on how you think it will affect the prospect of scaling up the national DMPA-SC program? What do you believe that the program should do in response to the influence of [state the most influential contextual factor]?*

[**NOTE TO INTERVIEWER:** FOR THE LATER COMPONENT OF 1.8, PROBE FOR WAYS IN WHICH THE DESIGN ELEMENTS OF THE PROGRAM MAY NEED TO CHANGE, LEADERSHIP DECISIONS THAT SHOULD BE MADE, MANAGEMENT STRATEGIES FOR MITIGATING THE ADVERSE EFFECTS AND/OR LEVERAGING THE POSITIVE EFFECT OF FACTORS].

1. **PROGRAMMATIC FEATURES AND IMPLEMENTATION STRATEGIES**
   1. *Reflecting on the progress of the national DMPA-SC program to date, what would you say are the positive outcomes of it that you have observed to date?*
   2. *What are the disappointing outcomes you have observed to date?*
   3. *Reflect on the design elements of the national program that you discussed earlier. Which of these are responsible for the positive outcomes you just mentioned? Why do you think these elements are responsible for these outcomes?*

[**NOTE TO INTERVIEWER:** SUCCINTLY SUMMARIZE THE PROGRAMMATIC DESIGN ELEMENTS THAT THE KEY INFORMANT DISCUSSED IN Q1.1. THE KEY INFORMANT CAN MENTION OTHER DESIGN ELEMENTS THAT S/HE DID NOT DISCUSS IN Q1.1 IF THEY WISH TO DO SO. IF NECESSARY TO ELICIT A RESPONSE, YOU CAN STATE THIS TO THE KEY INFORMANT. USE PROBES AND FOLLOW UP TO ELICIT RESPONSES FROM THE KEY INFORMANT FOR AS MANY OF THE POSITIVE OUTCOMES AS POSSIBLE].

- 1. *Consider the prospect of scaling up the national program on DMPA-SC in light of the information you just shared. Which of these program design elements should remain the same during scale up? Which of them should be adapted to enhance scale up?*
  2. *Consider the information you just shared on the program design elements that you believe must be adapted to enhance scale up. Why should these be adapted? What adaptations and changes should be made to these design elements?*
  3. *Reflect on the design elements of the national program that you discussed earlier. Which of these are responsible for the disappointing outcomes you just mentioned? Why do you think that these elements are responsible for these outcomes?*

[**NOTE TO INTERVIEWER:** THE KEY INFORMANT CAN MENTION OTHER DESIGN ELEMENTS THAT S/HE DID NOT DISCUSS IN Q1.1 IF THEY WISH TO DO SO. IF NECESSARY TO ELICIT A RESPONSE, YOU CAN STATE THIS TO THE KEY INFORMANT. USE PROBES AND FOLLOW UP TO ELICIT RESPONSES FROM THE KEY INFORMANT FOR AS MANY OF THE DISAPPOINTING OUTCOMES AS POSSIBLE].

- 1. *Consider the prospect of scaling up the national program on DMPA-SC in light of the information you just shared. Which of these program design elements should be eliminated during scale up? Which of them should be adapted to enhance scale up?*
  2. *Consider the information you just shared on the program design elements that you believe must be adapted to enhance scale up. Why should these be adapted? What adaptations and changes should be made to these design elements?*

***[AFTER THE KEY INFORMANT HAS RESPONDED TO 2.8, READ ALOUD – INTERVIEWERS CAN PARAPHRASE]:*** *That was our final question. Thank you for participating in this interview. At this time, do you have any further comments or information that you wish to share?*

[**NOTE TO INTERVIEWER:** IF THE KEY INFORMANT SAYS YES, ENCOURAGE HIM/HER TO SHARE HIS/HER COMMENTS. CONTINUE RECORDING THE INTERVIEW.

***[READ ALOUD]:*** *Do you have any questions at this time?*

[**NOTE TO INTERVIEWER:** IF THE KEY INFORMANT SAYS YES, ENCOURAGE HIM/HER TO ASK QUESTIONS. ANSWER THEM TO THE BEST OF YOUR ABILITY. IF YOU CANNOT ANSWER THEM, ENSURE THAT THESE QUESTIONS ARE NOTED AND REPLY THAT YOU WILL DO YOU BEST TO OBTAIN ANSWERS AND REPORT FEEDBCK TO THE KEY INFORMANT].

***[READ ALOUD]:*** *At this point, I believe we can end this interview. Thank you again.*

**Interviewer or note taker should turn of the digital recorder, record the time at which the interview ends, and depart, leaving with the key informant a business card that includes contact information for the study.**

END TIME OF IDI: ______________________
