# Supplementary material for: Enhancing the Introduction and Scale Up of Self-Administered Injectable Contraception (DMPA-SC) in Health Systems (the EASIER Project): Protocol for Embedded Implementation Research
Source: JMIR Res Protoc. 2023 Aug 23;12:e44222. doi: 10.2196/44222 (PMC10483301; doi:10.2196/44222)
Supplement: Multimedia Appendix 3 [file resprot_v12i1e44222_app3.doc]

## Instrument 3: Self-Administered DMPA-SC Program: Coverage and Structural

## Readiness Assessment

District Profile

| Country: |
| --- |
| District: |
| Number of public health care facilities in district: ___ referral hospitals ___ other tertiary facilities  ___ intermediate facilities ___ primary healthcare facilities  Number of private sector healthcare venues in district: ___ healthcare facilities ___ drug shops/pharmacies  District population:   |  |  |  |  |  |  | | --- | --- | --- | --- | --- | --- |   Number of communities in district: ___ population >5,000 ___ population 3,000-4,999  ___ population 1,000-2,999 ___ population <= 999 |
| Names and job title of members of District Health Management Team and DHMT role: |
| Questionnaire respondents and job title: |
| Date questionnaire completed: ____________________________________________________________________________________________________________ |

***INSTRUCTIONS:***

*The purpose of this questionnaire is to compile information on the coverage and structural readiness of the district to implement the national program of DMPA-SC for self-administration. Key informants should be public health system authorities within the local government apparatus that are responsible and experienced with the management of family planning services in the district. The questionnaire calls upon key informants to provide accurate information on the status of dissemination and use of knowledge and guidelines on the national program of DMPA-SC for self-administration at the district-level, and the general availability of DMPA-SC services (for self-administration) at the different levels of care in the local health system. It also inquires as to the availability of other contraceptive services that might be desired by DMPA-SC clients at some point during their course of using this method.*

*More than one, or even several, key informants, can participate in completing this questionnaire. To answer most of these questions, key informants are expected to consult available management documentation and records (e.g. comprehensive district health plans, budgets, supervision and health information system reports, training records, etc.). In addition, key informants should consult their counterparts from the different levels of care (e.g., community-based health care workers, primary-, intermediate- and tertiary-level facilities in-charges, private sector healthcare providers) about the status of the availability of DMPA-SC services for self-administration and supportive family planning services. At the end of the questionnaire, key informants should record the documents and records they used to obtain the information they report in this questionnaire. As well, they should record the other individuals they consulted to obtain the information they report in this questionnaire.*

*Below, key informants can record general comments they would like to report on their experience completing this form. These may include challenges, potential inaccuracies of the data they report, suggestions on additional information that would be pertinent to report, their perceptions of the relevance of the questions in this questionnaire, and those questions that could not be answered for lack of available information.*

| **Comments** |
| --- |
|  |

SECTION 1. DISTRICT-LEVEL DMPA-SC PROGRAM

*These questions should be directed to the district-level staff person(s) most knowledgeable on family planning related matters. Most questions should be answered by circling 1 for “Yes,” or 0 for “No.” Some questions have several pre-coded answers (see No. 1.4 as an example). In these cases, please circle the number next to the answer given. If the respondent does not give one of the pre-coded answers, circle the number next to “Other” and specify the answer in the space provided. Some questions ask the respondent to write estimates for certain measures of interest.*

| **No.** | **Item** | **Response** | **Skip to** |
| --- | --- | --- | --- |
| 1.1. | Has the district received any orientation or guidance on the national program of DMPA-SC for self-administration? | Yes 1  No 0 | If “No,” skip to 1.3 |
| 1.2 | Does this orientation or guidance include in person meetings with a representative of the national program from the Ministry of Health or other national partner? | Yes 1  *Who:* ____________________________  No 0 |  |
| 1.3 | Has the district received any written guidance, including materials about the national program on DMPA-SC for self-administration? | Yes 1  No 0 | If “No,” skip to 1.5 |
| 1.4 | Do these written materials include or involve any of the following *(circle all that apply)* | Yes No  Clinical standards and guidelines 1 0  Training curricula and materials 1 0  Supervision, quality assurance, client support 1 0  Community outreach and role of community members 1 0  Commodity security, supply chain logistics 1 0  Cost, insurance, social protections 1 0  IEC materials, job aids 1 0  Health information monitoring, reporting and systems 1 0  Planning and budgeting for DMPA-SC in district plans 1 0  Client rights to quality and comprehensive information 1 0  family planning information and services  Other: _______________________________________________________ |  |
| 1.5 | Is DMPA-SC for self-administration featured in district management tools or plans? | Yes 1  No 0 | If “No,” skip to 1.7 |
| 1.6 | In which district management tools and/or plans is DMPA-SC featured?  *(circle all that apply)* | Yes No  Budgets 1 0  Annual plans 1 0  Re-order and re-supply documents 1 0  Supervision forms 1 0  Service statistics aggregation forms/tools 1 0  Data base for managing information on  service utilization 1 0  Training (on the job, central) curricula, materials 1 0  Community-based health care related tool/materials 1 0  Other (write): _________________________________________________ __ |  |
| 1.7 | In addition to the public sector healthcare program, are there additional types of programs that deliver DMPA-SC for self-administration? | Yes 1  No 0 | If no skip to 1.10 |
| 1.8 | What are these programs? Do the district public healthcare authorities have a formal relationship with them?  *(circle all that apply)* | **Program in district Relationship**  Yes No Yes No  Pharmacies/drug shops 1 0 1 0  Private sector (e.g. religious) facilities 1 0 1 0  Non-governmental organizations 1 0 1 0  Community-based distribution 1 0 1 0 |  |
| 1.9 | How many of the following type of other types of programs that deliver DMPA-SC for self-administration are in your district? *(estimate for all that apply)* | Pharmacies/drug shops (no. pharmacies/shops) *write number:* ___________  Private sector (e.g. religious) facilities *write number:* ___________  Non-governmental organizations *write number:* ___________  Community-based distribution *write number:* ___________ |  |
| 1.10 | Does community-based distribution of DMPA-SC (for self-administration)take place in your district | Yes 1  *(estimate the number of communities in which CBD of DMPA-SC takes place):*  ______________________________________________________________.  No 0 |  |
| 1.11 | In this district, where are clients supposed to go to initiate use of DMPA-SC (for self-administration)?  *(circle all that apply)* | Yes No  Community-based health care worker 1 0  Primary healthcare facility 1 0  Intermediate health care facility 1 0  Tertiary healthcare facility 1 0  Private sector healthcare facility 1 0  Other: _______________________________________________________ |  |
| 1.12 | In this district, where are self-administering clients supposed to go to receive DMPA-SC related services after initiation? | Yes No  Community-based health care worker 1 0  Primary healthcare facility 1 0  Intermediate health care facility 1 0  Tertiary healthcare facility 1 0  Private sector healthcare facility 1 0  Other: _________________________________________________________ |  |

SECTION 2. DISTRICT BACKGROUND INFORMATION ON DMPA-SC AVAILABILITY AND UTILIZATION

*In this section, key informants should obtain the support from counterparts from facilities at different levels of care where family planning services are available in their district to answer the below questions.*

| 2.1 | ***Out of f all the points at which clients can receive DMPA-SC services (initiation for self-administration and post-initiation services), at how many can clients receive the following FP methods (NB: For A enter information on DMPA-SC; for B-K follow instructions).*** | | | | | | | | | | | | | |
| --- | --- | --- | --- | --- | --- | --- | --- | --- | --- | --- | --- | --- | --- | --- |
| **No.** | **FP Services** | **Community-based health care worker (write no. communities)** | | **Primary Healthcare Facility** | **Intermediate Healthcare Facility** | | **Tertiary Healthcare facility** | | **Private Sector Healthcare Facility** | | **Other: _________ _______________** | | **Other: _________ _______________** | |
| A | DMPA-SC | Write number: ____ | |  |  | |  | |  | |  | |  | |
| B | DMPA-IM | Write number: ____ | |  |  | |  | |  | |  | |  | |
| C | Oral contraceptives | Write number: ____ | |  |  | |  | |  | |  | |  | |
| D | Male condoms | Write number: ____ | |  |  | |  | |  | |  | |  | |
| E | Female condoms | Write number: ____ | |  |  | |  | |  | |  | |  | |
| F | Implants | Write number: ____ | |  |  | |  | |  | |  | |  | |
| G | Intra-uterine device | Write number: ____ | |  |  | |  | |  | |  | |  | |
| H | Tubal ligation | Write number: ____ | |  |  | |  | |  | |  | |  | |
| I | Vasectomy | Write number: ____ | |  |  | |  | |  | |  | |  | |
| J | Emergency contraception | Write number: ____ | |  |  | |  | |  | |  | |  | |
| K | Other methods: _______ | Write number: ____ | |  |  | |  | |  | |  | |  | |
| 2.2 | ***Of all the points at which clients can receive DMPA-SC services (initiation for self-administration and post-initiation services), how many healthcare workers are trained in providing the following methods (for none enter ‘0’, one to two, ‘1’, three to four, ‘2’, five to six, ‘3’, six to seven, ‘5’ seven or more, ‘6’). (NB: For A enter information on DMPA-SC; for B-K follow instructions).*** | | | | | | | | | | | | | |
| **No.** | **FP Services** | **Community-based health care worker (write no. communities)** | **Primary Healthcare Facility** | | | **Intermediate Healthcare Facility** | | **Tertiary Healthcare facility** | | **Private Sector Healthcare Facility** | | **Other: _________ _______________** | | **Other: _________ _______________** |
| A | DMPA-SC |  |  | | |  | |  | |  | |  | |  |
| B | DMPA-IM |  |  | | |  | |  | |  | |  | |  |
| C | Oral contraceptives |  |  | | |  | |  | |  | |  | |  |
| D | Male condoms |  |  | | |  | |  | |  | |  | |  |
| E | Female condoms |  |  | | |  | |  | |  | |  | |  |
| F | Implants |  |  | | |  | |  | |  | |  | |  |
| G | Intra-uterine device |  |  | | |  | |  | |  | |  | |  |
| H | Tubal ligation |  |  | | |  | |  | |  | |  | |  |
| I | Vasectomy |  |  | | |  | |  | |  | |  | |  |
| J | Emergency contraception |  |  | | |  | |  | |  | |  | |  |
| K | Other methods: _______ |  |  | | |  | |  | |  | |  | |  |
| 2.3 | ***Of all the points at which clients can receive DMPA-SC services (initiation for self-administration and post-initiation services), what is the median, minimum and maximum number of clients for the following family planning services (past 6 months)? (NB: For A enter information on DMPA-SC; for B-K follow instructions).*** | | | | | | | | | | | | | |
| **No.** | **FP Services** | **Community-based health care worker (write no. communities)** | | **Primary Healthcare Facility** | **Intermediate Healthcare Facility** | | **Tertiary Healthcare facility** | | **Private Sector Healthcare Facility** | | **Other: _________ _______________** | | **Other: _________ _______________** | |
| A | DMPA-SC |  | |  |  | |  | |  | |  | |  | |
| B | DMPA-IM |  | |  |  | |  | |  | |  | |  | |
| C | Oral contraceptives |  | |  |  | |  | |  | |  | |  | |
| D | Male condoms |  | |  |  | |  | |  | |  | |  | |
| E | Female condoms |  | |  |  | |  | |  | |  | |  | |
| F | Implants |  | |  |  | |  | |  | |  | |  | |
| G | Intra-uterine device |  | |  |  | |  | |  | |  | |  | |
| H | Tubal ligation |  | |  |  | |  | |  | |  | |  | |
| I | Vasectomy |  | |  |  | |  | |  | |  | |  | |
| J | Emergency contraception |  | |  |  | |  | |  | |  | |  | |
| K | Other methods: _______ |  | |  |  | |  | |  | |  | |  | |
| 2.4 | ***Of all the points at which clients can receive DMPA-SC services (initiation for self-administration and post-initiation services), how many have experienced stock outs of supplies or commodities essential for the following family planning services (past 6 months). (NB: For A enter information on DMPA-SC; for B-K follow instructions) ?*** | | | | | | | | | | | | | |
| **No.** | **FP Services** | **Community-based health care worker (write no. communities)** | | **Primary Healthcare Facility** | **Intermediate Healthcare Facility** | | **Tertiary Healthcare facility** | | **Private Sector Healthcare Facility** | | **Other: _________ _______________** | | **Other: _________ _______________** | |
| A | DMPA-SC | Write number: | |  |  | |  | |  | |  | |  | |
| B | DMPA-IM |  | |  |  | |  | |  | |  | |  | |
| C | Oral contraceptives |  | |  |  | |  | |  | |  | |  | |
| D | Male condoms |  | |  |  | |  | |  | |  | |  | |
| E | Female condoms |  | |  |  | |  | |  | |  | |  | |
| F | Implants |  | |  |  | |  | |  | |  | |  | |
| G | Intra-uterine device |  | |  |  | |  | |  | |  | |  | |
| H | Tubal ligation |  | |  |  | |  | |  | |  | |  | |
| I | Vasectomy |  | |  |  | |  | |  | |  | |  | |
| J | Emergency contraception |  | |  |  | |  | |  | |  | |  | |
| K | Other methods: _______ |  | |  |  | |  | |  | |  | |  | |
| 2.5 | ***Do staff from these groups of health workers provide the following FP services? (Provision of services should reflect real circumstances and not whether the health worker is authorized or formally trained to provide the care. Exclude health workers who only assist. Ask the questions only if there is someone on staff in each available group.)*** | | | | | | | | | | | | | |

| **No.** | **FP services** | **a. Medical doctors (GP, ob/gyn)** | **b. Other clinician** | **c. Midwives** | **d. Nurses** | **e. Other non-clinician healthcare worker** | **f. Community Health Worker** | **e. to be specified** |
| --- | --- | --- | --- | --- | --- | --- | --- | --- |
| A | DMPA-SC | Yes 1  No 0 | Yes 1  No 0 | Yes 1  No 0 | Yes 1  No 0 | Yes 1  No 0 | Yes 1  No 0 | Yes 1  No 0 |
| B | DMPA-IM | Yes 1  No 0 | Yes 1  No 0 | Yes 1  No 0 | Yes 1  No 0 | Yes 1  No 0 | Yes 1  No 0 | Yes 1  No 0 |
| C | Oral contraceptives | Yes 1  No 0 | Yes 1  No 0 | Yes 1  No 0 | Yes 1  No 0 | Yes 1  No 0 | Yes 1  No 0 | Yes 1  No 0 |
| D | Male condoms | Yes 1  No 0 | Yes 1  No 0 | Yes 1  No 0 | Yes 1  No 0 | Yes 1  No 0 | Yes 1  No 0 | Yes 1  No 0 |
| E | Female condoms | Yes 1  No 0 | Yes 1  No 0 | Yes 1  No 0 | Yes 1  No 0 | Yes 1  No 0 | Yes 1  No 0 | Yes 1  No 0 |
| F | Emergency contraception | Yes 1  No 0 | Yes 1  No 0 | Yes 1  No 0 | Yes 1  No 0 | Yes 1  No 0 | Yes 1  No 0 | Yes 1  No 0 |
| G | Implant | Yes 1  No 0 | Yes 1  No 0 | Yes 1  No 0 | Yes 1  No 0 | Yes 1  No 0 | Yes 1  No 0 | Yes 1  No 0 |
| H | Intra-uterine device | Yes 1  No 0 | Yes 1  No 0 | Yes 1  No 0 | Yes 1  No 0 | Yes 1  No 0 | Yes 1  No 0 | Yes 1  No 0 |
| I | Tubal ligation | Yes 1  No 0 | Yes 1  No 0 | Yes 1  No 0 | Yes 1  No 0 | Yes 1  No 0 | Yes 1  No 0 | Yes 1  No 0 |
| J | Vasectomy | Yes 1  No 0 | Yes 1  No 0 | Yes 1  No 0 | Yes 1  No 0 | Yes 1  No 0 | Yes 1  No 0 | Yes 1  No 0 |
| K | Other: _________ | Yes 1  No 0 | Yes 1  No 0 | Yes 1  No 0 | Yes 1  No 0 | Yes 1  No 0 | Yes 1  No 0 | Yes 1  No 0 |

SECTION 3. INFORMATION SOURCES

*In this section, key informants should report on the sources of information they consulted in order to obtain their responses to the items in this questionnaire. Sources of information can be management documentation and records, as well as colleagues that also work in the district healthcare system.*

| **No.** | **Item** | **Information source** | **Comments** |
| --- | --- | --- | --- |
| 1.1. | Has the district received any orientation or guidance on the national program of DMPA-SC for self-administration? |  |  |
| 1.2 | Does this orientation or guidance include in person meetings with a representative of the national program from the Ministry of Health or other national partner? |  |  |
| 1.3 | Has the district received any written guidance, including materials about the national program on DMPA-SC for self-administration? |  |  |
| 1.4 | Do these written materials include or involve any of the following *(circle all that apply)* |  |  |
| 1.5 | Is DMPA-SC for self-administration featured in district management tools or plans? |  |  |
| 1.6 | In which district management tools and/or plans is DMPA-SC featured? |  |  |
| 1.7 | In addition to the public sector healthcare program, are there additional types of programs that deliver DMPA-SC for self-administration? |  |  |
| 1.8 | What are these programs? Do the district public healthcare authorities have a formal relationship with them? |  |  |
| 1.9 | How many of the following type of other types of programs that deliver DMPA-SC for self-administration are in your district? *(estimate for all that apply)* |  |  |
| 1.10 | Does community-based distribution of DMPA-SC (for self-administration)take place in your district |  |  |
| 1.11 | In this district, where are clients supposed to go to initiate use of DMPA-SC (for self-administration)? |  |  |
| 1.12 | In this district, where are self-administering clients supposed to go to receive DMPA-SC related services after initiation? |  |  |
| 2.1 | Of all the points at which clients can receive DMPA-SC services (initiation for self-administration and post-initiation services), at how many can clients receive the following FP methods (see above). |  |  |
| 2.2 | Of all the points at which clients can receive DMPA-SC services (initiation for self-administration and post-initiation services), how many healthcare workers are trained in providing the following family planning methods (see above). |  |  |
| 2.3 | Of all the points at which clients can receive DMPA-SC services (initiation for self-administration and post-initiation services), what is the median, minimum and maximum number of clients for the following family planning services (past 6 months)? (See above). |  |  |
| 2.4 | Of all the points at which clients can receive DMPA-SC services (initiation for self-administration and post-initiation services), how many have experienced stock outs of supplies or commodities essential for the following family planning services (past 6 months)? (See above). |  |  |
| 2.5 | Do staff from these groups of health workers provide the following FP services? (Provision of services should reflect real circumstances and not whether the health worker is authorized or formally trained to provide the care. Exclude health workers who only assist. Ask the questions only if there is someone on staff in each available group.) (See above). |  |  |
