# Supplementary material for: Enhancing the Introduction and Scale Up of Self-Administered Injectable Contraception (DMPA-SC) in Health Systems (the EASIER Project): Protocol for Embedded Implementation Research
Source: JMIR Res Protoc. 2023 Aug 23;12:e44222. doi: 10.2196/44222 (PMC10483301; doi:10.2196/44222)
Supplement: Multimedia Appendix 4 [file resprot_v12i1e44222_app4.doc]

## Instrument 4: Self-Administered DMPA-SC Program: Organizational Readiness for

## Change Assessment

District Profile

| Country: |
| --- |
| District: |
| Names, job title and place of work of respondents: |
| Date questionnaire completed: |

***INSTRUCTIONS:***

*The purpose of this questionnaire is to compile information on the organizational readiness of your district to carry out the changes required by the national program of DMPA-SC for self-administration. Key informants should be public health system authorities within the local government apparatus that are responsible and experienced with the management of family planning services in the district. They can also be facilities’ in charges that are, or will be, responsible for managing the DMPA-SC program at the facility level. The questionnaire calls upon key informants to report their perceptions of the evidence base regarding DMPA-SC for self-administration and its relevance to the settings in which they work. It also asks key informants to share their assessment of the feasibility for implementing the changes called for by the national program, taking into account the levels of leadership and implementation capacity where they work. Lastly, it asks key informants to provide their assessment of the level of commitment and efficacy to implement the changes required for this program.*

*The purpose of this assessment is to obtain strategic information on the readiness to adopt the program so that national program planners and technical assistance organizers understand the needs that must be met in order that introduction of and/or support for the program in each district is planned appropriately. Each district is asked to provide one form per district. The completed form should reflect the consensus of local experts, like those mentioned in the paragraph above, who have discussed and agreed on best responses to the items of the questionnaire below.*

*Below, key informants can record general comments they would like to report on their experience completing this form. This may include challenges, potential inaccuracies of the data reported, suggestions on additional information that would be pertinent to report, their perceptions of the relevance of the questions in this questionnaire, and those questions that could not be answered for lack of available information.*

| **Comments** |
| --- |
|  |

SECTION 1: EVIDENCE ASSESSMENT

*1.1 Credibility of Evidence*

*Based on your assessment of the evidence basis for the following statement, please rate the strength of evidence in your opinion and based on how you think respected experts in the district feel about the strength of evidence. Please do so on a scale of 1 to 5, where 1 is very weak evidence and 5 is very strong evidence.*

| **No.** | **Item** | **Rank** | | | | | |
| --- | --- | --- | --- | --- | --- | --- | --- |
| ***The availability of DMPA-SC for self-injection will improve contraceptive use in my district.*** | | | | | | | |
| **1.1.1** | Based on your assessment of the evidence basis for this statement, please rate the strength of the evidence in your opinion. | **Very weak (1)** | **Weak (2)** | **Neither weak nor strong (3)** | **Strong (4)** | **Very strong (5)** | **Don’t know (not applicable) (99)** |
| **1.1.2** | Now, please do so based on how you think respected experts in the district feel about the strength of the evidence. | **Very weak (1)** | **Weak (2)** | **Neither weak nor strong (3)** | **Strong (4)** | **Very strong (5)** | **Don’t know (not applicable (99)** |
| ***The availability of DMPA-SC for self-injection, overall, makes work easier for family planning providers in my district.*** | | | | | | | |
| **1.1.3** | Based on your assessment of the evidence basis for this statement, please rate the strength of the evidence in your opinion. | **Very weak (1)** | **Weak (2)** | **Neither weak nor strong (3)** | **Strong (4)** | **Very strong (5)** | **Don’t know (not applicable (99)** |
| **1.1.4** | Now, please do so based on how you think respected experts in the district feel about the strength of the evidence. | **Very weak (1)** | **Weak (2)** | **Neither weak nor strong (3)** | **Strong (4)** | **Very strong (5)** | **Don’t know (not applicable (99)** |
| ***The majority of family planning clients in my district prefer to be able to administer DMPA injections independently than to receive it from a healthcare provider.*** | | | | | | | |
| **1.1.5** | Based on your assessment of the evidence basis for this statement, please rate the strength of the evidence in your opinion. | **Very weak (1)** | **Weak (2)** | **Neither weak nor strong (3)** | **Strong (4)** | **Very strong (5)** | **Don’t know (not applicable (99)** |
| **1.1.6** | Now, please do so based on how you think respected experts in the district feel about the strength of the evidence. | **Very weak (1)** | **Weak (2)** | **Neither weak nor strong (3)** | **Strong (4)** | **Very strong (5)** | **Don’t know (not applicable (99)** |
| ***The availability of DMPA-SC for self-administration, from the health system perspective, makes family planning provision more affordable.*** | | | | | | | |
| **1.1.7** | Based on your assessment of the evidence basis for this statement, please rate the strength of the evidence in your opinion. | **Very weak (1)** | **Weak (2)** | **Neither weak nor strong (3)** | **Strong (4)** | **Very strong (5)** | **Don’t know (not applicable (99)** |
| **1.1.8** | Now, please do so based on how you think respected experts in the district feel about the strength of the evidence. | **Very weak (1)** | **Weak (2)** | **Neither weak nor strong (3)** | **Strong (4)** | **Very strong (5)** | **Don’t know (not applicable (99)** |
| ***The availability of DMPA-SC for self-administration, from the client perspective, makes family planning provision more affordable.*** | | | | | | | |
| **1.1.9** | Based on your assessment of the evidence basis for this statement, please rate the strength of the evidence in your opinion. | **Very weak (1)** | **Weak (2)** | **Neither weak nor strong (3)** | **Strong (4)** | **Very strong (5)** | **Don’t know (not applicable (99)** |
| **1.1.10** | Now, do so based on how you think respected experts in the district feel about the strength of the evidence. | **Very weak (1)** | **Weak (2)** | **Neither weak nor strong (3)** | **Strong (4)** | **Very strong (5)** | **Don’t know (not applicable (99)** |
| ***Community-based distribution of DMPA-SC is a relatively effective way to make this method most accessible to women in this district.*** | | | | | | | |
| **1.1.11** | Based on your assessment of the evidence basis for this statement, please rate the strength of the evidence in your opinion. | **Very weak (1)** | **Weak (2)** | **Neither weak nor strong (3)** | **Strong (4)** | **Very strong (5)** | **Don’t know (not applicable (99)** |
| **1.1.12** | Now, do so based on how you think respected experts in the district feel about the strength of the evidence. | **Very weak (1)** | **Weak (2)** | **Neither weak nor strong (3)** | **Strong (4)** | **Very strong (5)** | **Don’t know (not applicable (99)** |
| ***Self-injection of DMPA-SC is a safe way for women to meet their family planning needs.*** | | | | | | | |
| **1.1.13** | Based on your assessment of the evidence basis for this statement, please rate the strength of the evidence in your opinion. | **Very weak (1)** | **Weak (2)** | **Neither weak nor strong (3)** | **Strong (4)** | **Very strong (5)** | **Don’t know (not applicable (99)** |
| **1.1.14** | Now, please do so based on how you think respected experts in the district feel about the strength of the evidence. | **Very weak (1)** | **Weak (2)** | **Neither weak nor strong (3)** | **Strong (4)** | **Very strong (5)** | **Don’t know (not applicable (99)** |

*1.2 Relevance of Evidence*

*Based on your experience working on family planning in this district, please rate your level of agreement with the following statements on the relevance of available evidence on self-administered DMPA-SC vis-à-vis the prospect of implementing DMPA-SC for self-administration through the structures and with the resources of the local healthcare system in your district. Please do so on a scale of 1 to 5, where 1 is strongly disagree and 5 is strongly agree.*

| **No.** | **Item** | **Rank** | | | | | |
| --- | --- | --- | --- | --- | --- | --- | --- |
| ***Implementation of a program on DMPA-SC for self-administration in our district requires changes that…*** | | | | | | | |
| **1.2.1** | Are supported by evidence that comes from this district. | **Strongly disagree (1)** | **Disagree (2)** | **Neither agree or disagree (3)** | **Agree (4)** | **Strongly agree (5)** | **Don’t know (not applicable) (99)** |
| **1.2.2** | Are supported by evidence from external settings that are equivalent to our district. | **Strongly disagree (1)** | **Disagree (2)** | **Neither agree or disagree (3)** | **Agree (4)** | **Strongly agree (5)** | **Don’t know (not applicable) (99)** |
| **1.2.3** | Are supported by evidence that applies well to the context of our district health care system. | **Strongly disagree (1)** | **Disagree (2)** | **Neither agree or disagree (3)** | **Agree (4)** | **Strongly agree (5)** | **Don’t know (not applicable) (99)** |
| **1.2.4** | Should be effective in this district based on current knowledge about this program. | **Strongly disagree (1)** | **Disagree (2)** | **Neither agree or disagree (3)** | **Agree (4)** | **Strongly agree (5)** | **Don’t know (not applicable) (99)** |
| **1.2.5** | Are supported by our district’s healthcare workers clinical experience with family planning clients. | **Strongly disagree (1)** | **Disagree (2)** | **Neither agree or disagree (3)** | **Agree (4)** | **Strongly agree (5)** | **Don’t know (not applicable) (99)** |
| **1.2.6** | Are supported by our district’s community-based healthcare workers’/teams’ experience with family planning clients. | **Strongly disagree (1)** | **Disagree (2)** | **Neither agree or disagree (3)** | **Agree (4)** | **Strongly agree (5)** | **Don’t know (not applicable) (99)** |
| **1.2.7** | Conform to the opinions and attitudes of family planning providers and other experts in this district. | **Strongly disagree (1)** | **Disagree (2)** | **Neither agree or disagree (3)** | **Agree (4)** | **Strongly agree (5)** | **Don’t know (not applicable) (99)** |
| **1.2.8** | Conform to the opinions and attitudes of community-based healthcare workers/teams in this district. | **Strongly disagree (1)** | **Disagree (2)** | **Neither agree or disagree (3)** | **Agree (4)** | **Strongly agree (5)** | **Don’t know (not applicable) (99)** |
| **1.2.9** | Have been well-accepted by family planning clients in a pilot study in our district | **Strongly disagree (1)** | **Disagree (2)** | **Neither agree or disagree (3)** | **Agree (4)** | **Strongly agree (5)** | **Don’t know (not applicable) (99)** |
| **1.2.10** | Are consistent with practices that have been accepted by family planning clients in our district. | **Strongly disagree (1)** | **Disagree (2)** | **Neither agree or disagree (3)** | **Agree (4)** | **Strongly agree (5)** | **Don’t know (not applicable) (99)** |
| **1.2.11** | Take into consideration the needs and preferences of family planning clients in this district. | **Strongly disagree (1)** | **Disagree (2)** | **Neither agree or disagree (3)** | **Agree (4)** | **Strongly agree (5)** | **Don’t know (not applicable) (99)** |
| **1.2.12** | Appear to have more advantages than disadvantages for family planning clients in this district. | **Strongly disagree (1)** | **Disagree (2)** | **Neither agree or disagree (3)** | **Agree (4)** | **Strongly agree (5)** | **Don’t know (not applicable) (99)** |

SECTION 2: FEASIBILITY ASSESSMENT

*2.1 Leadership and Management*

*Based on your experience working on family planning in this district, please rate your level of agreement with the following statements about leadership and management characteristics in the local context of implementing the* national program on self-administered DMPA-SC has been and/or will be implemented. Please do so on a scale of 1 to 5, where 1 is strongly disagree and 5 is strongly agree.

| **No.** | **Item** | | **Rank** | | | | | |
| --- | --- | --- | --- | --- | --- | --- | --- | --- |
| ***Leadership in administrative, clinical management and community outreach settings in your district healthcare system…*** | | | | | | | | |
| **2.1.1** | Rewards innovation and creativity to improve the implementation of the self-administered DMPA-SC program. | | **Strongly disagree (1)** | **Disagree (2)** | **Neither agree or disagree (3)** | **Agree (4)** | **Strongly agree (5)** | **Don’t know (not applicable) (99)** |
| **2.1.2** | Solicits the opinions and ideas of staff regarding decisions about how to improve the self-administered DMPA-SC program. | | **Strongly disagree (1)** | **Disagree (2)** | **Neither agree or disagree (3)** | **Agree (4)** | **Strongly agree (5)** | **Don’t know (not applicable) (99)** |
| **2.1.3** | Seeks ways to improve client satisfaction and increase client participation in the self-administered DMPA-SC program. | | **Strongly disagree (1)** | **Disagree (2)** | **Neither agree or disagree (3)** | **Agree (4)** | **Strongly agree (5)** | **Don’t know (not applicable) (99)** |
| **2.1.4** | Seeks ways to improve community acceptance and increase community involvement in the self-administered DMPA-SC program. | | **Strongly disagree (1)** | **Disagree (2)** | **Neither agree or disagree (3)** | **Agree (4)** | **Strongly agree (5)** | **Don’t know (not applicable) (99)** |
| **2.1.5** | Provides effective management for continuous improvement of client care. | | **Strongly disagree (1)** | **Disagree (2)** | **Neither agree or disagree (3)** | **Agree (4)** | **Strongly agree (5)** | **Don’t know (not applicable) (99)** |
| **2.1.6** | Clearly defines areas of responsibility and authority for staff at different levels of care. | | **Strongly disagree (1)** | **Disagree (2)** | **Neither agree or disagree (3)** | **Agree (4)** | **Strongly agree (5)** | **Don’t know (not applicable) (99)** |
| **2.1.7** | Promotes team building within and between levels of care to address challenges in client care. | | **Strongly disagree (1)** | **Disagree (2)** | **Neither agree or disagree (3)** | **Agree (4)** | **Strongly agree (5)** | **Don’t know (not applicable) (99)** |
| **2.1.8** | Promotes communication within and between levels of care to address challenges in client care. | | **Strongly disagree (1)** | **Disagree (2)** | **Neither agree or disagree (3)** | **Agree (4)** | **Strongly agree (5)** | **Don’t know (not applicable) (99)** |
| **2.1.9** | Provides staff with information on performance measures, guidelines and implementation plans. | | **Strongly disagree (1)** | **Disagree (2)** | **Neither agree or disagree (3)** | **Agree (4)** | **Strongly agree (5)** | **Don’t know (not applicable) (99)** |
| **2.1.10** | Establishes clear goals for process and outcomes related to client care and rights. | | **Strongly disagree (1)** | **Disagree (2)** | **Neither agree or disagree (3)** | **Agree (4)** | **Strongly agree (5)** | **Don’t know (not applicable) (99)** |
| **2.1.11** | Establishes clear goals for processes and outcomes related to service coverage and accessibility. | | **Strongly disagree (1)** | **Disagree (2)** | **Neither agree or disagree (3)** | **Agree (4)** | **Strongly agree (5)** | **Don’t know (not applicable) (99)** |
| **2.1.12** | Provides staff members with feedback on the effects of individual and team performance. | | **Strongly disagree (1)** | **Disagree (2)** | **Neither agree or disagree (3)** | **Agree (4)** | **Strongly agree (5)** | **Don’t know (not applicable) (99)** |
| **2.1.13** | Holds staff accountable for achieving results. | | **Strongly disagree (1)** | **Disagree (2)** | **Neither agree or disagree (3)** | **Agree (4)** | **Strongly agree (5)** | **Don’t know (not applicable) (99)** |
| ***Staff members in in your district healthcare system…*** | | | | | | | | |
| **2.1.14** | Have a sense of personal responsibility for improving care and outcomes for family planning clients. | | **Strongly disagree (1)** | **Disagree (2)** | **Neither agree or disagree (3)** | **Agree (4)** | **Strongly agree (5)** | **Don’t know (not applicable) (99)** |
| **2.1.15** | Cooperate to maintain and improve effectiveness of care to family planning clients. | | **Strongly disagree (1)** | **Disagree (2)** | **Neither agree or disagree (3)** | **Agree (4)** | **Strongly agree (5)** | **Don’t know (not applicable) (99)** |
| **2.1.16** | Cooperate with counterparts at different places where family planning information and services are provided to improve effectiveness of care to family planning clients. | | **Strongly disagree (1)** | **Disagree (2)** | **Neither agree or disagree (3)** | **Agree (4)** | **Strongly agree (5)** | **Don’t know (not applicable) (99)** |
| **2.1.17** | Are willing to innovate and/or experiment to improve the quality and accessibility of family planning information and services. | | **Strongly disagree (1)** | **Disagree (2)** | **Neither agree or disagree (3)** | **Agree (4)** | **Strongly agree (5)** | **Don’t know (not applicable) (99)** |
| **2.1.18** | Are willing to try new service delivery protocols and processes, even if this requires addition duties and responsibilities. | | **Strongly disagree (1)** | **Disagree (2)** | **Neither agree or disagree (3)** | **Agree (4)** | **Strongly agree (5)** | **Don’t know (not applicable) (99)** |
| ***Leadership in the local government in your district…*** | | | | | | | | |
| **2.1.19** | | Seeks ways to ensure the quality and acceptability of the self-administered DMPA-SC program. | **Strongly disagree (1)** | **Disagree (2)** | **Neither agree or disagree (3)** | **Agree (4)** | **Strongly agree (5)** | **Don’t know (not applicable) (99)** |
| **2.1.20** | | Seeks ways to ensure the sustainability of the self-administered DMPA-SC program. | **Strongly disagree (1)** | **Disagree (2)** | **Neither agree or disagree (3)** | **Agree (4)** | **Strongly agree (5)** | **Don’t know (not applicable) (99)** |
| **2.1.21** | | Seeks ways to expand the coverage and accessibility of the self-administered DMPA-SC program. | **Strongly disagree (1)** | **Disagree (2)** | **Neither agree or disagree (3)** | **Agree (4)** | **Strongly agree (5)** | **Don’t know (not applicable) (99)** |
| ***Community-level leadership in your district…*** | | | | | | | | |
| **2.1.22** | | Seeks ways to ensure the quality and acceptability of the self-administered DMPA-SC program. | **Strongly disagree (1)** | **Disagree (2)** | **Neither agree or disagree (3)** | **Agree (4)** | **Strongly agree (5)** | **Don’t know (not applicable) (99)** |
| **2.1.23** | | Seeks ways to ensure the sustainability of the self-administered DMPA-SC program. | **Strongly disagree (1)** | **Disagree (2)** | **Neither agree or disagree (3)** | **Agree (4)** | **Strongly agree (5)** | **Don’t know (not applicable) (99)** |
| **2.1.24** | | Seeks ways to expand the coverage and accessibility of the self-administered DMPA-SC program. | **Strongly disagree (1)** | **Disagree (2)** | **Neither agree or disagree (3)** | **Agree (4)** | **Strongly agree (5)** | **Don’t know (not applicable) (99)** |
| **2.1.25** | | Seeks ways to strengthen linkages and collaboration with the formal health system (district-management and facilities). | **Strongly disagree (1)** | **Disagree (2)** | **Neither agree or disagree (3)** | **Agree (4)** | **Strongly agree (5)** | **Don’t know (not applicable) (99)** |

*2.2 Implementation Capacity*

*Based on your experience working on family planning in this district, please rate your level of agreement with the following statements about the capacity of the healthcare system in your district to implement the national program on self-adm*inistered DMPA-SC has been and/or will be implemented. Please do so on a scale of 1 to 5, where 1 is strongly disagree and 5 is strongly agree.

| **No.** | **Item** | **Rank** | | | | | |
| --- | --- | --- | --- | --- | --- | --- | --- |
| ***The clinical and supervisory capacity of the district healthcare system is strong enough to…*** | | | | | | | |
| **2.2.1** | Meet the demand for training and task sharing responsibilities at all levels of care where the DMPA-SC program in implemented. | **Strongly disagree (1)** | **Disagree (2)** | **Neither agree or disagree (3)** | **Agree (4)** | **Strongly agree (5)** | **Don’t know (not applicable) (99)** |
| **2.2.2** | Ensure that clients have access to essential information and education, including written materials and follow up visits, about DMPA-SC continuously where they live. | **Strongly disagree (1)** | **Disagree (2)** | **Neither agree or disagree (3)** | **Agree (4)** | **Strongly agree (5)** | **Don’t know (not applicable) (99)** |
| **2.2.3** | Ensure that DMPA-SC clients with adverse events receive timely and technically sound clinical attention. | **Strongly disagree (1)** | **Disagree (2)** | **Neither agree or disagree (3)** | **Agree (4)** | **Strongly agree (5)** | **Don’t know (not applicable) (99)** |
| **2.2.4** | Ensure that clients have access to a wide range of contraceptive methods if and when they decide to change methods. | **Strongly disagree (1)** | **Disagree (2)** | **Neither agree or disagree (3)** | **Agree (4)** | **Strongly agree (5)** | **Don’t know (not applicable) (99)** |
| **2.2.5** | Regulate and document adherence to service delivery guidelines on self-administered DMPA-SC. | **Strongly disagree (1)** | **Disagree (2)** | **Neither agree or disagree (3)** | **Agree (4)** | **Strongly agree (5)** | **Don’t know (not applicable) (99)** |
| **2.2.6** | Ensure a timely response to lapses in adherence to service delivery guidelines and that corrective actions are effective. | **Strongly disagree (1)** | **Disagree (2)** | **Neither agree or disagree (3)** | **Agree (4)** | **Strongly agree (5)** | **Don’t know (not applicable) (99)** |
| ***The commodity security and logistics capacity of the district healthcare system is strong enough to…*** | | | | | | | |
| **2.2.7** | Ensure that DMPA-SC supplies and commodities are always available when clients may need them. | **Strongly disagree (1)** | **Disagree (2)** | **Neither agree or disagree (3)** | **Agree (4)** | **Strongly agree (5)** | **Don’t know (not applicable) (99)** |
| **2.2.8** | Ensure the safe storage and disposal of DMPA-SC commodities where clients use them. | **Strongly disagree (1)** | **Disagree (2)** | **Neither agree or disagree (3)** | **Agree (4)** | **Strongly agree (5)** | **Don’t know (not applicable) (99)** |
| **2.2.9** | Ensure that DMPA-SC clients desiring to switch methods can conveniently do so at all times. | **Strongly disagree (1)** | **Disagree (2)** | **Neither agree or disagree (3)** | **Agree (4)** | **Strongly agree (5)** | **Don’t know (not applicable) (99)** |
| ***The financial capacity of the district healthcare system is strong enough to…*** | | | | | | | |
| **2.2.10** | Ensure that DMPA-SC and other family planning commodities are always available where they are needed. | **Strongly disagree (1)** | **Disagree (2)** | **Neither agree or disagree (3)** | **Agree (4)** | **Strongly agree (5)** | **Don’t know (not applicable) (99)** |
| **2.2.11** | Ensure that community-based elements of the DMPA-SC program take place routinely and well. | **Strongly disagree (1)** | **Disagree (2)** | **Neither agree or disagree (3)** | **Agree (4)** | **Strongly agree (5)** | **Don’t know (not applicable) (99)** |
| **2.2.12** | Ensure that the clinical and training elements of the DMPA-SC program take place routinely and well. | **Strongly disagree (1)** | **Disagree (2)** | **Neither agree or disagree (3)** | **Agree (4)** | **Strongly agree (5)** | **Don’t know (not applicable) (99)** |
| **2.2.13** | Ensure that eligible clients and households are identified and receive social protections allowed by policy (e.g. fee exemptions, enrollment in social or community insurance schemes). | **Strongly disagree (1)** | **Disagree (2)** | **Neither agree or disagree (3)** | **Agree (4)** | **Strongly agree (5)** | **Don’t know (not applicable) (99)** |
| **2.2.14** | Ensure that all essential user fee and cost recovery procedures are implemented effectively and according to established procedures | **Strongly disagree (1)** | **Disagree (2)** | **Neither agree or disagree (3)** | **Agree (4)** | **Strongly agree (5)** | **Don’t know (not applicable) (99)** |
| ***The health information recording and monitoring processes of the district healthcare system is strong enough to…*** | | | | | | | |
| **2.2.15** | Integrate data on DMPA-SC self-administration into existing data collection and reporting tools and ensure that providers record these well. | **Strongly disagree (1)** | **Disagree (2)** | **Neither agree or disagree (3)** | **Agree (4)** | **Strongly agree (5)** | **Don’t know (not applicable) (99)** |
| **2.2.16** | Ensure that data on DMPA-SC self-administration are routinely reported into a repository where they are stored systematically. | **Strongly disagree (1)** | **Disagree (2)** | **Neither agree or disagree (3)** | **Agree (4)** | **Strongly agree (5)** | **Don’t know (not applicable) (99)** |
| **2.2.17** | Routinely guide performance improvement and management decision-making with data on DMPA-SC self-administration. | **Strongly disagree (1)** | **Disagree (2)** | **Neither agree or disagree (3)** | **Agree (4)** | **Strongly agree (5)** | **Don’t know (not applicable) (99)** |

SECTION 3: COMMITMENT AND EFFICACY ASSESSMENT

*3.1 Program facilitation*

*Based on your experience working on family planning in this district, please rate your level of agreement with the following statements about the commitment to facilitate implementation of the self-administered DMPA-SC program districts health system. Please do so on a scale of 1 to 5, where 1 is strongly disagre*e and 5 is strongly agree.

| **No.** | **Item** | **Rank** | | | | | |
| --- | --- | --- | --- | --- | --- | --- | --- |
| ***Leadership of self-administered DMPA-SC program in the district will…*** | | | | | | | |
| **3.1.1** | Propose a project and implementation plan that is appropriate and feasible. | **Strongly disagree (1)** | **Disagree (2)** | **Neither agree or disagree (3)** | **Agree (4)** | **Strongly agree (5)** | **Don’t know (not applicable) (99)** |
| **3.1.2** | Provide clear goals for implementation and performance improvement. | **Strongly disagree (1)** | **Disagree (2)** | **Neither agree or disagree (3)** | **Agree (4)** | **Strongly agree (5)** | **Don’t know (not applicable) (99)** |
| **3.1.3** | Ensure adequate resources for accomplishing the self-administered DMPA-SC project and implementation plan. | **Strongly disagree (1)** | **Disagree (2)** | **Neither agree or disagree (3)** | **Agree (4)** | **Strongly agree (5)** | **Don’t know (not applicable) (99)** |
| **3.1.4** | Set a high priority on the success of the self-administered DMPA-SC. | **Strongly disagree (1)** | **Disagree (2)** | **Neither agree or disagree (3)** | **Agree (4)** | **Strongly agree (5)** | **Don’t know (not applicable) (99)** |
| **3.1.5** | Facilitate communication between team members at all levels of care where the self-administered DMPA-SC is implemented. | **Strongly disagree (1)** | **Disagree (2)** | **Neither agree or disagree (3)** | **Agree (4)** | **Strongly agree (5)** | **Don’t know (not applicable) (99)** |
| **3.1.6** | Ensure regular feedback to team members on the progress of the self-administered DMPA-SC. | **Strongly disagree (1)** | **Disagree (2)** | **Neither agree or disagree (3)** | **Agree (4)** | **Strongly agree (5)** | **Don’t know (not applicable) (99)** |
| **3.1.7** | Ensure regular feedback to team members at all levels of care on the strength of their performance. | **Strongly disagree (1)** | **Disagree (2)** | **Neither agree or disagree (3)** | **Agree (4)** | **Strongly agree (5)** | **Don’t know (not applicable) (99)** |
| **3.1.8** | Use data from the self-administered DMPA-SC program to evaluate the program and make management decisions. | **Strongly disagree (1)** | **Disagree (2)** | **Neither agree or disagree (3)** | **Agree (4)** | **Strongly agree (5)** | **Don’t know (not applicable) (99)** |
| **3.1.9** | Incentivize high quality performance through recognition, rewards, feedback and distribution of performance measures. | **Strongly disagree (1)** | **Disagree (2)** | **Neither agree or disagree (3)** | **Agree (4)** | **Strongly agree (5)** | **Don’t know (not applicable) (99)** |
| ***Implementation team members of self-administered DMPA-SC program in the district will…*** | | | | | | | |
| **3.1.10** | Share responsibility for the success of the self-administered DMPA-SC program. | **Strongly disagree (1)** | **Disagree (2)** | **Neither agree or disagree (3)** | **Agree (4)** | **Strongly agree (5)** | **Don’t know (not applicable) (99)** |
| **3.1.11** | Have clearly defined roles, tasks, responsibilities and timelines. | **Strongly disagree (1)** | **Disagree (2)** | **Neither agree or disagree (3)** | **Agree (4)** | **Strongly agree (5)** | **Don’t know (not applicable) (99)** |
| **3.1.12** | Have adequate time to accomplish their tasks and responsibilities on time and within their regular workload. | **Strongly disagree (1)** | **Disagree (2)** | **Neither agree or disagree (3)** | **Agree (4)** | **Strongly agree (5)** | **Don’t know (not applicable) (99)** |
| **3.1.13** | Be continuously incentivized to perform their roles up to appropriate standards. | **Strongly disagree (1)** | **Disagree (2)** | **Neither agree or disagree (3)** | **Agree (4)** | **Strongly agree (5)** | **Don’t know (not applicable) (99)** |
| **3.1.14** | Sustain personal motivation to perform their roles up to appropriate standards. | **Strongly disagree (1)** | **Disagree (2)** | **Neither agree or disagree (3)** | **Agree (4)** | **Strongly agree (5)** | **Don’t know (not applicable) (99)** |

*3.2 Efficacy to implement the program*

*Based on your experience working on family planning in this district, and reflecting on the aspects of implementation capacity discussed in Section 2.1, report your overall perceptions of the efficacy of your district to implement the self-administered DMPA-SC program. Please do so by reporting for level of agreement with the following statements of implementation efficacy on a scale of 1 to 5, where 1 is strongly disagree and 5 is strongly agree.*

| **No.** | **Item** | **Rank** | | | | | |
| --- | --- | --- | --- | --- | --- | --- | --- |
| ***Overall, our district has the potential to achieve outstanding implementation of…*** | | | | | | | |
| **3.2.1** | The training, task sharing, supervision and clinical aspects of the self-administered DMPA-SC program. | **Strongly disagree (1)** | **Disagree (2)** | **Neither agree or disagree (3)** | **Agree (4)** | **Strongly agree (5)** | **Don’t know (not applicable) (99)** |
| **3.2.2** | The supply chain, commodity security and logistics aspects of the self-administered DMPA-SC program. | **Strongly disagree (1)** | **Disagree (2)** | **Neither agree or disagree (3)** | **Agree (4)** | **Strongly agree (5)** | **Don’t know (not applicable) (99)** |
| **3.2.3** | The financial and pro-equity aspects of the self-administered DMPA-SC program. | **Strongly disagree (1)** | **Disagree (2)** | **Neither agree or disagree (3)** | **Agree (4)** | **Strongly agree (5)** | **Don’t know (not applicable) (99)** |
| **3.2.4** | The health information aspects of the self-administered DMPA-SC program. | **Strongly disagree (1)** | **Disagree (2)** | **Neither agree or disagree (3)** | **Agree (4)** | **Strongly agree (5)** | **Don’t know (not applicable) (99)** |
| **3.2.5** | The aspects of the program on clients’ rights to continuous access to information and services. | **Strongly disagree (1)** | **Disagree (2)** | **Neither agree or disagree (3)** | **Agree (4)** | **Strongly agree (5)** | **Don’t know (not applicable) (99)** |
| **3.2.6** | The aspects of the program on community involvement and support to community-based members of the implementation team. | **Strongly disagree (1)** | **Disagree (2)** | **Neither agree or disagree (3)** | **Agree (4)** | **Strongly agree (5)** | **Don’t know (not applicable) (99)** |
| **3.2.7** | The management requirements to ensure that the program has the resources it needs to succeed. | **Strongly disagree (1)** | **Disagree (2)** | **Neither agree or disagree (3)** | **Agree (4)** | **Strongly agree (5)** | **Don’t know (not applicable) (99)** |
| **3.2.8** | The management requirements to ensure that the district receives the technical assistants it needs to succeed. | **Strongly disagree (1)** | **Disagree (2)** | **Neither agree or disagree (3)** | **Agree (4)** | **Strongly agree (5)** | **Don’t know (not applicable) (99)** |
