# Supplementary material for: Enhancing the Introduction and Scale Up of Self-Administered Injectable Contraception (DMPA-SC) in Health Systems (the EASIER Project): Protocol for Embedded Implementation Research
Source: JMIR Res Protoc. 2023 Aug 23;12:e44222. doi: 10.2196/44222 (PMC10483301; doi:10.2196/44222)
Supplement: Multimedia Appendix 7 [file resprot_v12i1e44222_app7.docx]

**Subnational-level Focus Group Discussion: Contextual Influences on, and Programmatic Determinants of the Outcomes of the Local DMPA-SC Program.**

**Instructions:**

- This focus group discussion (FGD) is intended to obtain strategic information from key informants on (a) factors that influence the effectiveness, feasibility and sustainability of the DMPA-SC program, and (b) the features of the program, and how it has been implemented, that have shaped these outcomes of the program.
- Focus groups should comprise of participants that come from subnational-level organizations involved in the DMPA-SC program (e.g. District Health Management Teams, Healthcare Workers, members of Community-based structures, private sector and NGO staff, traditional or local political leaders). There should be distinct focus groups for each different type of participant. In all groups, participants should describe the local-level influences in response to objective (a) above. Participants should draw upon their perspectives as those that have helped implement the DMPA-SC program in their locality to respond to objective (b).
- For both objectives of the FGD, participants should elucidate these factors and features, how they operate and why they affect the effectiveness, feasibility and sustainability of the DMPA-SC program. They should explain why and how they believe these factors and features will influence the scale up of the DMPA-SC program in their locality (e.g. scale up within a district, or from one district to a neighboring district).
- For questions concerning objective (a), participants should describe how they think the DMPA-SC program needs to react in order to mitigate adverse contextual influences and leverage positive contextual influences.
- For questions concerning objective (b), participants should describe the salient features of the program, and implementation strategies they have used, that have shaped the outcomes of the DMPA-SC program. They should explain if they believe these features and strategies should be continued during scale up, eliminated and why? They should describe if they should be adapted as the DMPA-SC program goes to scale, and, if so, how and why.

| Name of data collector:  ___________________________________________________________________________________________  Date:  ___________________________________________________________________________________________  Country and District:  ___________________________________________________________________________________________  Location of the interview:  ___________________________________________________________________________________________  Name of FGD Member 1:  ___________________________________________________________________________________________  Job Title FGD Member 1:  ___________________________________________________________________________________________  Organization where FGD Member 1 is employed: ___________________________________________________________________________________________  Number of Years FGD Member 1 has been in that job: ___________________________________________________________________________________________  Role of FGD Member 1 in the Local DMPA-SC Program:  **___________________________________________________________________________________________**  Name of FGD Member 2:  ___________________________________________________________________________________________  Job Title FGD Member 2:  ___________________________________________________________________________________________  Organization where FGD Member 2 is employed: ___________________________________________________________________________________________  Number of Years FGD Member 2 has been in that job: ___________________________________________________________________________________________  Role of FGD Member 2 in the Local DMPA-SC Program:  ___________________________________________________________________________________________  Name of FGD Member 3:  ___________________________________________________________________________________________  Job Title FGD Member 3:  ___________________________________________________________________________________________  Organization where FGD Member 3 is employed: ___________________________________________________________________________________________  Number of Years FGD Member 3 has been in that job: ___________________________________________________________________________________________  Role of FGD Member 3 in the Local DMPA-SC Program:  Name of FGD Member 4:  ___________________________________________________________________________________________  Job Title FGD Member 4:  ___________________________________________________________________________________________  Organization where FGD Member 4 is employed: ___________________________________________________________________________________________  Number of Years FGD Member 4 has been in that job: ___________________________________________________________________________________________  Role of FGD Member 4 in the Local DMPA-SC Program:  ___________________________________________________________________________________________  Name of FGD Member 5:  ___________________________________________________________________________________________  Job Title FGD Member 5:  ___________________________________________________________________________________________  Organization where FGD Member 5 is employed: ___________________________________________________________________________________________  Number of Years FGD Member 5 has been in that job: ___________________________________________________________________________________________  Role of FGD Member 5 in the Local DMPA-SC Program:  ___________________________________________________________________________________________  Name of FGD Member 6:  ___________________________________________________________________________________________  Job Title FGD Member 6:  ___________________________________________________________________________________________  Organization where FGD Member 6 is employed: ___________________________________________________________________________________________  Number of Years FGD Member 6 has been in that job: ___________________________________________________________________________________________  Role of FGD Member 6 in the Local DMPA-SC Program:  ___________________________________________________________________________________________ |
| --- |

**Introduction:**

**FACILITATOR SHOULD CONFIRM WITH THE FGD PARTICIPANTS THAT S/HE HAS PERMISSION TO RECORD THE FGD ON A RECORDED DEVICE. IF THE PARTICIPANTS CONSENT, THE INTERVIEWER CAN START RECORDING NOW. RECORD THE TIME AT WHICH THE INTERVIEW BEGINS.**

START TIME OF FGD: ______________________

***[READ ALOUD – FACILITATORS CAN PARAPHRASE]:*** *Thank you for taking the time to participate in this focus group discussion on the DMPA-SC program in [name of district^[[1]](#footnote-1)^]. As you are aware, the policies of [name of country] permit the use of DMPA-SC for self-administration. Women who desire the method, can be screened by a healthcare worker and, provided that they are eligible for the method, receive their first injection at the facility together with a re-supply kit of DMPA which they can inject into themselves, sub-cutaneously, at home after their initial injection has expired. As you are aware, this program is being implemented in your district. We are interested in your perceptions of the DMPA-SC program, as it is being carried out in your district. In particular, we are interested in your views on the factors in the local environment – e.g. district, local health system or community contexts – which you are familiar with in your role as [state the job titles of the participants] at [state the participants’ places of work], that influence the program, its effectiveness, its feasibility and its sustainability. Secondly, we are interested your perception of the features of the program of DMPA-SC in your district that you believe are responsible for the outcomes of the program – the program’s effectiveness, feasibility and sustainability. In addition, if you are able to comment on this, we would like to know your perceptions of how program implementers deliver the program, and how you think implementation approaches have led to the program’s outcomes to date. Finally, reflecting on the factors and programmatic features you discuss, we would like your views on what the program needs to do in the future as it pursues scale up.*

**Discussion:**

1. **CONTEXTUAL INFLUENCES ON THE LOCAL DMPA-SC PROGRAM**
   1. *Let us think about our knowledge and experience with this program in [name of district], and brainstorm different elements of the design of the local program of self-administered DMPA-SC in your district. In other words, based on your understanding, how is the DMPA-SC program supposed to operate in your district?*

[**NOTE TO FACILITATOR:** IF NECESSARY, SUGGEST YOU MEAN BY ‘ELEMENTS OF THE DESIGN OF THE LOCAL PROGRAM:

- HEALTH WORKER ROLES
- TRAINING AND TASK SHIFTING
- QUALITY ASSURANCE AND SUPERVISION
- CLIENT FOLLOW UP AND CONTINUING SUPPORT
- LOGISTICS, SUPPLY CHAIN AND COMMODITY SECURITY, RESUPPLY AND DISTRIBUTION
- ROLES OF THE COMMUNITY LEADERS, TEAMS/COMMITTEES, COMMUNITY HEALTH WORKERS, VOLUNTEERS
- HOW THE DISTRICT PROGRAM IS FINANCED/BUDGETED
- COST RECOVERY/USER FEES
- MONITORING, INDICATORS AND HEALTH INFORMATION SYSTEMS
- ENROLLING USERS IN INSURANCE, IDENTIFYING THOSE ELIGIBLE FOR FEE EXEMPTIONS AND SOCIAL PROTECTIONS
- NATIONAL POLICY, STANDARDS DISSEMINATION AT LOCAL LEVEL
- DISTRICT PROGRAM MANAGEMENT AND COORDINATION STRUCTURES
- ORGANIZATIONAL AND LEADERSHIP ROLES (e.g. DISTRICT HEALTH MANAGEMENT TEAMS, ROLES OF DIFFERENT MEMBERS, NGO/TECHNICAL ASSISTANCE PARTNERS)
- OTHER SECTORS INVOLVED IN THE DISTRICT PROGRAM AND THEIR ROLES

PARTICIPANTS DO **NOT** HAVE TO PROVIDE INFORMATION ON ALL ASPECTS OF THE PROGRAM; HOWEVER PROBE REPEATEDLY, BUT SENSITIVELY, TO ELICIT AS MUCH INFORMATION AS REALISTICALLY POSSIBLE.]

- 1. *As I mentioned in the introduction, we would like to understand your perceptions of factors in the local environment that influence the program – positively and negatively. These influences include local politics, health system management issues, how the health system works with community- or traditional-structures, cultural and social relations and communication networks. Reflecting on the elements of the design of the DMPA-SC program in your district, let us take a moment to brainstorm the factors that influence if these design elements can be successful.*

[NOTE TO FACILITATOR: IF NECESSARY, DESCRIBE SPECIFIC TYPES OF FACTORS, AND PROMPT PARTICIPANTS TO EXPLAIN WHAT THESE ARE IN RELATION TO THE INDIVIDUAL FACTORS MENTIONED IN RESPONSE TO Q1.1. SPECIFIC TYPES OF FACTORS ARE:

- LEADERSHIP (E.G. CHANGES IN LEADERSHIP, OPINIONS, POSITIONS, AND ATTITUDES OF CURRENT LEADERS, E.G. POLITICAL, RELIGIOUS, HEALTH SYSTEM, COMMUNITY, ETC).
- FINANCIAL FACTORS (BUDGETING, FLOWS OF REVENUE THAT SUSTAIN THE LOCAL HEALTH SYSTEM).
- TRAINING AND SUPERVISION CAPACITY, WORKFORCE AVAILABILITY AT DIFFERENT LEVELS OF CARE
- MANAGEMENT CAPACITY VIS-À-VIS DEMANDS OF EXTENDING LOCAL HEALTH SYSTEM TO HOUSEHOLDS WHERE USERS SELF-INJECT
- HOW NATIONAL POLICIES AND STANDARDS ARE DISSEMINATED AT THE LOCAL LEVEL
- LOCAL PROCUREMENT AND DISTRIBUTION PROCESS (CHANGES, DISRUPTIONS)
- INFLUENCES OF COMMUNITY POLITICAL AND HEALTH CARE STRUCTURES
- SOCIAL RELATIONS, NETWORKS AND NORMS
- CULTURAL, RELIGIOUS INFLUENCES
- LOCAL ECONOMIC CONDITIONS
- COMMUNICATION NETWORKS IN THE DISTRICT
- OTHER EMERGING PUBLIC HEALTH PRIORITIES IN THE DISTRICT

THIS QUESTION ASKS FOR BRAINSTORMING OF FACTORS IN RELATION TO DESIGN ELEMENTS MENTIONED IN Q1.1, NOT EXHAUSTIVE EXPLANATION OF HOW THE FACTORS INFLUENCE THE PROGRAM. PARTICIPANTS DO **NOT** HAVE TO PROVIDE INFORMATION ON ALL POTENTIAL CONTEXTUAL FACTORS. MAKE SURE THAT YOU OR THE IDI NOTETAKER DOCUMENTS THE FACTORS MENTIONED AND THE CORRESPONDING PROGRAM DESIGN ELEMENT.]

- 1. *Just to confirm, you all have identified the following contextual factors affecting the following elements of the local program of DMPA-SC.*

[**NOTE TO FACILITATOR:** SUMMARIZE ALOUD, SUCCINCTLY, THE CONTEXTUAL FACTORS MENTIONED IN Q1.2 AND THE CORRESPONDING PROGRAM DESIGN ELEMENTS MENTIONED IN Q1.1]

*As a group, can you rank in the order of most influential to least influential the three most influential contextual factors you discussed moments ago? Explain your justification for ranking these as you do.*

- 1. *Reflecting on the most influential contextual factor, can you all elaborate on how it affects the outcomes of the program that you have observed to date?*

[**NOTE TO FACILITATOR:** PROBE SPECIFICALLY FOR PARTICIPANTS’ PERCEPTIONS OF THE INFLUENCE OF FACTORS ON OUTCOMES ON EFFECTIVENESS, FEASIBILITY TO DELIVER THE INTERVENTION THROUGH THE LOCAL PUBLIC HEALTH SYSTEM, SUSTAINABILITY OF THE LOCAL PROGRAM].

- 1. *Reflecting on the second most influential contextual factor, can you all elaborate on how it affects the outcomes of the program that you have observed to date?*

[**NOTE TO FACILITATOR:** PROBE SPECIFICALLY FOR PARTICIPANTS’ PERCEPTIONS OF THE INFLUENCE OF FACTORS ON OUTCOMES ON EFFECTIVENESS, FEASIBILITY TO DELIVER THE INTERVENTION THROUGH THE LOCAL PUBLIC HEALTH SYSTEM, SUSTAINABILITY OF THE LOCAL PROGRAM].

- 1. *Reflecting on the third most influential contextual factor, can you all elaborate on how it affects the outcomes of the program that you have observed to date?*

[**NOTE TO FACILITATOR:** PROBE SPECIFICALLY FOR PARTICIPANTS’ PERCEPTIONS OF THE INFLUENCE OF FACTORS ON OUTCOMES ON EFFECTIVENESS, FEASIBILITY TO DELIVER THE INTERVENTION THROUGH THE LOCAL PUBLIC HEALTH SYSTEM, SUSTAINABILITY OF THE LOCAL PROGRAM].

- 1. *Reflecting on the most influential contextual factor, can you all elaborate on how you think it will affect the prospect of scaling up the DMPA-SC program in your district?^[[2]](#footnote-2)^ What do you believe that the program should do in response to the influence of [state the most influential contextual factor]?*

[**NOTE TO FACILITATOR:** FOR THE LATER COMPONENT OF 1.7, PROBE FOR WAYS IN WHICH THE DESIGN ELEMENTS OF THE PROGRAM MAY NEED TO CHANGE, LEADERSHIP DECISIONS THAT SHOULD BE MADE, MANAGEMENT STRATEGIES FOR MITIGATING THE ADVERSE EFFECTS AND/OR LEVERAGING THE POSITIVE EFFECT OF FACTORS].

- 1. *Reflecting on the second most influential contextual factor, can you all elaborate on how you think it will affect the prospect of scaling up the DMPA-SC program in your district?2 What do you believe that the program should do in response to the influence of [state the most influential contextual factor]?*

[**NOTE TO FACILITATOR:** FOR THE LATER COMPONENT OF 1.8, PROBE FOR WAYS IN WHICH THE DESIGN ELEMENTS OF THE PROGRAM MAY NEED TO CHANGE, LEADERSHIP DECISIONS THAT SHOULD BE MADE, MANAGEMENT STRATEGIES FOR MITIGATING THE ADVERSE EFFECTS AND/OR LEVERAGING THE POSITIVE EFFECT OF FACTORS].

- 1. *Reflecting on the third most influential contextual factor, can you all elaborate on how you think it will affect the prospect of scaling up the DMPA-SC program in your district?2 What do you believe that the program should do in response to the influence of [state the most influential contextual factor]?*

[**NOTE TO FACILITATOR:** FOR THE LATER COMPONENT OF 1.9, PROBE FOR WAYS IN WHICH THE DESIGN ELEMENTS OF THE PROGRAM MAY NEED TO CHANGE, LEADERSHIP DECISIONS THAT SHOULD BE MADE, MANAGEMENT STRATEGIES FOR MITIGATING THE ADVERSE EFFECTS AND/OR LEVERAGING THE POSITIVE EFFECT OF FACTORS].

1. **PROGRAMMATIC FEATURES AND IMPLEMENTATION STRATEGIES**
   1. *Reflecting on the progress of the DMPA-SC program [STATE NAME OF DISTRICT] to date, can we brainstorm the positive outcomes of it that you have observed to date?*
   2. *Now, can we brainstorm the disappointing outcomes you have observed to date?*
   3. *Let’s all think about the design elements of the DMPA-SC program in [STATE NAME OF DISTRICT] that you all discussed earlier. Which of these are responsible for the positive outcomes you just mentioned? Why do you think these elements are responsible for these outcomes?*

[**NOTE TO FACILITATOR:** SUCCINTLY SUMMARIZE THE PROGRAMMATIC DESIGN ELEMENTS THAT PARTICIPANTS DISCUSSED IN Q1.1. PARTICIPANTS CAN MENTION OTHER DESIGN ELEMENTS THAT THEY DID NOT DISCUSS IN Q1.1 IF THEY WISH TO DO SO. IF NECESSARY TO ELICIT A RESPONSE, YOU CAN STATE THIS TO THE KEY PARTICIPANTS. PROBE PARTICIPANTS FOR THE FOLLOWING:

- THE PEOPLE THAT WERE RESPONSIBLE FOR IMPLEMENTING THE EFFECTIVE DESIGN ELEMENTS
- HOW THEY IMPLEMENTED THOSE DESIGN ELEMENTS
- WHY THEIR IMPLEMENTATION STRATEGY OR APPROACH WORKED].
  1. *Consider the prospect of scaling up the DMPA-SC program in your district2 in light of the information we just discussed. Which of these program design elements do you all think should remain the same during scale up? Which of them do you all think should be adapted to enhance scale up?*
  2. *Consider the information we just discussed on the program design elements that you believe must be adapted to enhance scale up. Why do you all believe that these elements should be adapted? What adaptations and changes do you all think should be made to these design elements?*

[**NOTE TO FACILITATOR:** ALLOW PARTICIPANTS TO DESCRIBE HOW THEY THINK DESIGN ELEMENT SHOULD REMAIN AND/OR BE ADAPTED. THEN, PROBE PARTICIPANTS TO REFLECT ON THE INFORMATION THEY JUST SHARED ABOUT IMPLEMENTATION OF THE DESIGN ELEMENTS (E.G. PROBE OF 2.3), AND HOW IMPLEMENTATION STRATEGIES MIGHT BE CONTINUED OR ADAPTED].

- 1. *Let’s recall the design elements of the local program that you all discussed earlier. Which of these do you all believe are responsible for the disappointing outcomes we all just discussed? Why do you all think that these elements are responsible for these outcomes?*

[**NOTE TO FACITATOR:** PARTICIPANTS CAN MENTION OTHER DESIGN ELEMENTS THAT THEY DID NOT DISCUSS IN Q1.1 IF THEY WISH TO DO SO. IF NECESSARY TO ELICIT A RESPONSE, YOU CAN STATE THIS TO PARTICIPANTS. PROBE PARTICIPANTS FOR THE FOLLOWING:

- THE PEOPLE THAT WERE RESPONSIBLE FOR IMPLEMENTING THE INEFFECTIVE DESIGN ELEMENTS
- HOW THEY IMPLEMENTED THOSE DESIGN ELEMENTS
- WHY THEIR IMPLEMENTATION STRATEGY OR APPROACH WAS NOT EFFECTIVE].
  1. *Consider the prospect of scaling up the DMPA-SC program in your district2 in light of the information we just discussed. Which of these program design elements do you all believe should be eliminated during scale up? Which of them do you all believe should be adapted to enhance scale up?*
  2. *Consider the information we just discussed on the program design elements that you believe must be adapted to enhance scale up. Why do you all believe that should these be adapted? What adaptations and changes do you all believe should be made to these design elements?*

[**NOTE TO FACILITATOR:** ALLOW PARTICIPANTS TO DESCRIBE HOW THEY THINK DESIGN ELEMENT SHOULD REMAIN AND/OR BE ADAPTED. THEN, PROBE PARTICIPANTS TO REFLECT ON THE INFORMATION THEY JUST SHARED ABOUT IMPLEMENTATION OF THE DESIGN ELEMENTS (E.G. PROBE OF 2.6), AND HOW IMPLEMENTATION STRATEGIES MIGHT BE CONTINUED OR ADAPTED].

***[AFTER THE PARTICIPANTS HAVE RESPONDED TO 2.8, READ ALOUD – FACILITATORS CAN PARAPHRASE]:*** *That was our final question. Thank you for participating in this discussion. At this time, do you have any further comments or information that you wish to share?*

[**NOTE TO FACILITATOR:** IF A PARTICIPANT SAYS YES, ENCOURAGE HIM/HER TO SHARE HIS/HER COMMENTS. CONTINUE RECORDING THE INTERVIEW.

***[READ ALOUD]:*** *Do you have any questions at this time?*

[**NOTE TO FACILITATOR:** IF A PARTICIPANT SAYS YES, ENCOURAGE HIM/HER TO ASK QUESTIONS. ANSWER THEM TO THE BEST OF YOUR ABILITY. IF YOU CANNOT ANSWER THEM, ENSURE THAT THESE QUESTIONS ARE NOTED AND REPLY THAT YOU WILL DO YOU BEST TO OBTAIN ANSWERS AND REPORT FEEDBACK TO THE PARTICIPANT].

***[READ ALOUD]:*** *At this point, I believe we can end this interview. Thank you again.*

**Facilitator or note taker should turn of the digital recorder, record the time at which the interview ends, and depart, leaving with the key informant a business card that includes contact information for the study.**

END TIME OF FGD: ______________________

1. We use the term “district” here to refer to the sub-nationality localities in which local level scale up is occurring in the country where the implementation research is taking place. This may vary across countries (i.e. some countries have districts, others provinces, councils, etc.) [↑](#footnote-ref-1)
2. If scale up within the district is not realistic, then suggest that the Key Informant reflects on the prospect of scaling up from her/his district to a neighboring district where the program is not implemented. [↑](#footnote-ref-2)
