# Supplementary material for: Enhancing the Introduction and Scale Up of Self-Administered Injectable Contraception (DMPA-SC) in Health Systems (the EASIER Project): Protocol for Embedded Implementation Research
Source: JMIR Res Protoc. 2023 Aug 23;12:e44222. doi: 10.2196/44222 (PMC10483301; doi:10.2196/44222)
Supplement: Multimedia Appendix 8 [file resprot_v12i1e44222_app8.docx]

**Community-level In-depth Interview: Contextual Influences on, and Programmatic Determinants of the Outcomes of the Local DMPA-SC Program.**

**Instructions:**

- This focus group discussion (FGD) is intended to obtain strategic information from participants on (a) factors that influence the effectiveness, feasibility and sustainability of the DMPA-SC program, and (b) the features of the program, and how it has been implemented, that have shaped these outcomes of the program.
- Focus groups should comprise of participants that come from community-level organizations or structures involved in the DMPA-SC program (e.g. Healthcare Workers that conduct outreach, members of community governance structures and health committees, community health workers or volunteers, influential cultural figures in the community, representatives of women’s groups). There should be distinct focus groups for each different type of participant. In all groups, participants should describe the community-level influences in response to objective (a) above. Participants should draw upon their perspectives as those that have helped implement the DMPA-SC program in their community to respond to objective (b).
- For both objectives of the FGD, participants should elucidate these factors and features, how they operate and why they affect the effectiveness, feasibility and sustainability of the DMPA-SC program in their community. They should explain why and how they believe these factors and features will influence the scale up of the DMPA-SC program to other communities in their locality (e.g. district).
- For questions concerning objective (a), participants should describe how they think the DMPA-SC program needs to react in order to mitigate adverse contextual influences and leverage positive contextual influences.
- For questions concerning objective (b), participants should describe the features of the program, and implementation strategies they have used, that have shaped the outcomes of the program in their community. They should explain if they believe these features and strategies should be continued during scale up, eliminated and why? They should describe if they should be adapted as the DMPA-SC program goes to scale, and, if so, how and why.

| Name of data collector:  ___________________________________________________________________________________________  Date: ___________________________________________________________________________________________Country and District:  ___________________________________________________________________________________________  Location of the interview:  ___________________________________________________________________________________________  Name of FGD Member 1:  ___________________________________________________________________________________________  Job Title FGD Member 1:  ___________________________________________________________________________________________  Organization where FGD Member 1 is employed: ___________________________________________________________________________________________  Number of Years FGD Member 1 has been in that job: ___________________________________________________________________________________________  Role of FGD Member 1 in the Local DMPA-SC Program:  **___________________________________________________________________________________________**  Name of FGD Member 2:  ___________________________________________________________________________________________  Job Title FGD Member 2:  ___________________________________________________________________________________________  Organization where FGD Member 2 is employed: ___________________________________________________________________________________________  Number of Years FGD Member 2 has been in that job: ___________________________________________________________________________________________  Role of FGD Member 2 in the Local DMPA-SC Program:  ___________________________________________________________________________________________  Name of FGD Member 3:  ___________________________________________________________________________________________  Job Title FGD Member 3:  ___________________________________________________________________________________________  Organization where FGD Member 3 is employed: ___________________________________________________________________________________________  Number of Years FGD Member 3 has been in that job: ___________________________________________________________________________________________  Role of FGD Member 3 in the Local DMPA-SC Program:  Name of FGD Member 4:  ___________________________________________________________________________________________  Job Title FGD Member 4:  ___________________________________________________________________________________________  Organization where FGD Member 4 is employed: ___________________________________________________________________________________________  Number of Years FGD Member 4 has been in that job: ___________________________________________________________________________________________  Role of FGD Member 4 in the Local DMPA-SC Program:  ___________________________________________________________________________________________  Name of FGD Member 5:  ___________________________________________________________________________________________  Job Title FGD Member 5:  ___________________________________________________________________________________________  Organization where FGD Member 5 is employed: ___________________________________________________________________________________________  Number of Years FGD Member 5 has been in that job: ___________________________________________________________________________________________  Role of FGD Member 5 in the Local DMPA-SC Program:  ___________________________________________________________________________________________  Name of FGD Member 6:  ___________________________________________________________________________________________  Job Title FGD Member 6:  ___________________________________________________________________________________________  Organization where FGD Member 6 is employed: ___________________________________________________________________________________________  Number of Years FGD Member 6 has been in that job: ___________________________________________________________________________________________  Role of FGD Member 6 in the Local DMPA-SC Program:  ___________________________________________________________________________________________ |
| --- |

**Introduction:**

**FACILITATOR SHOULD CONFIRM WITH THE FGD PARTICIPANTS THAT S/HE HAS PERMISSION TO RECORD THE FGD ON A RECORDED DEVICE. IF THE PARTICIPANTS CONSENT, THE INTERVIEWER CAN START RECORDING NOW. RECORD THE TIME AT WHICH THE INTERVIEW BEGINS.**

START TIME OF FGD: ______________________

***[READ ALOUD – FACILITATORS CAN PARAPHRASE]:*** *Thank you for taking the time to participate in this focus group discussion on the DMPA-SC program in [name of community]. As you are aware, the policies of [name of country] permit the use of DMPA-SC for self-administration. Women who desire the method, can be screened by a healthcare worker and, provided that they are eligible for the method, receive their first injection at the facility together with a re-supply kit of DMPA which they can inject into themselves, sub-cutaneously, at home after their initial injection has expired. As you are aware, this program is being implemented in your community. We are interested in your perceptions of the DMPA-SC program, as it is being carried out in your community. In particular, we are interested in your view on the factors in the community environment – e.g. household, social groups, religious structures, community government other community groups. You are familiar with the factors in your role as [state the role of the key informant in the community DMPA-SC program] at [state the name of the community], and we would like to know how you think these factors influence the program in your community, its effectiveness, its feasibility and its sustainability. Secondly, we are interested your perception of the features of the program of DMPA-SC in your community that you believe are responsible for the outcomes of the program – the program’s effectiveness, feasibility and sustainability – in your community. In addition, if you are able to comment on this, we would like to know your perceptions of how program implementers deliver the program in your community, and how you think implementation approaches have led to the program’s outcomes to date. Finally, reflecting on the factors and programmatic features you discuss, we would like your views on what the program needs to do in the future as it pursues scale up.*

**Discussion:**

1. **CONTEXTUAL INFLUENCES ON THE COMMUNITY DMPA-SC PROGRAM**
   1. *Let us think about our knowledge and experience with this program in [name of community], and brainstorm different elements of the design of the program of self-administered DMPA-SC in your community. In other words, based on your understanding, how is the DMPA-SC program supposed to operate in your community?*

[**NOTE TO FACILITATOR:** IF NECESSARY, SUGGEST YOU MEAN BY ‘ELEMENTS OF THE DESIGN OF THE LOCAL PROGRAM:

- HEALTH WORKER ROLES IN COMMUNITY-BASED HEALTH CARE AND OUTREACH
- CLIENT FOLLOW UP AND SUPPORT, QUALITY ASSURANCE AND SUPERVISION IN THE COMMUNITY
- ROLE OF COMMUNITY GOVERNMENTS, HEALTH COMMITTEES, COMMUNITY HEALTH WORKERS, VOLUNTEERS IN IMPLEMENTING THE PROGRAM.
- ROLE OF WOMEN’S GROUPS, RELIGIOUS LEADERS, OTHER COMMUNITY ORGANIZATIONS
- TRAINING AND TASK SHARING IN THE COMMUNITY, OR OF COMMUNITY MEMBERS TO PARTICIPATE IN THE DMPA-SC PROGRAM
- LOGISTICS, SUPPLY CHAIN AND COMMODITY SECURITY, RESUPPLY AND DISTRIBUTION OF COMMODITIES IN THE COMMUNITY
- HOW THE COMMUNITY PROGRAM IS FINANCED/BUDGETED
- HEALTH INFORMATION COLLECTED IN THE COMMUNITY, MONITORING AND REPORTED
- ENROLLING USERS IN INSURANCE, IDENTIFYING THOSE ELIGIBLE FOR FEE EXEMPTIONS AND SOCIAL PROTECTIONS
- COMMUNITY PROGRAM MANAGEMENT AND COORDINATION STRUCTURES
- TECHNICAL ASSISTANCE PARTNERS (E.G. NGO) OTHER SECTORS INVOLVED IN THE DISTRICT PROGRAM AND THEIR ROLES

PARTICIPANTS DO **NOT** HAVE TO PROVIDE INFORMATION ON ALL ASPECTS OF THE PROGRAM; HOWEVER PROBE REPEATEDLY, BUT SENSITIVELY, TO ELICIT AS MUCH INFORMATION AS REALISTICALLY POSSIBLE.]

- 1. *As I mentioned in the introduction, we would like to understand your perceptions of factors in the community environment that influence the program – positively and negatively. These influences include community politics and policies, interactions and relationships with the formal health system, social relations and networks, cultural and religious norms, and communication networks. Reflecting on the elements of the design of the DMPA-SC program in your community, let us take a moment to brainstorm the factors that influence if these design elements can be successful.*

[NOTE TO FACILITATOR: IF NECESSARY, DESCRIBE THE SPECIFIC TYPES OF FACTORS AND PROMPT PARTICIPANTS TO DESCRIBE WHAT THESE ARE IN RELATION TO THE INDIVIDUAL FACTORS MENTIONED IN RESPONSE TO Q1.1. SPECIFIC TYPES OF FACTORS ARE:

- LEADERSHIP IN THE COMMUNITY (E.G. CHANGES IN LEADERSHIP, OPINIONS, POSITIONS, AND ATTITUDES OF POLITICAL, RELIGIOUS, SOCIAL LEADERS).
- ECONOMIC CONDITIONS IN THE COMMUNITY, HOW THESE EFFECT USE OF COMMUNITY RESOURCES FOR HEALTHCARE
- PARTNERSHIP WITH THE FORMAL HEALTH SYSTEM ON TRAINING, SUPERVISION, QUALITY ASSURANCE, CLIENT FOLLOW UP AND SUPPORT
- CHARACTERISTICS AND CAPACITY OF COMMUNITY HEALTH WORKERS AND VOLUNTEERS
- COMMUNITY-BASED HEALTHCARE MANAGEMENT CAPACITY VIS-À-VIS DEMANDS OF DMPA-SC PROGRAM IN COMMUNITY
- DISSEMINATION OF NATIONAL POLICIES AND STANDARDS AT THE COMMUNITY LEVEL
- SUPPLY CHAIN LOGISTICS, COMMODITY SECURITY AND DISTRIBUTION IN THE COMMUNITY
- SOCIAL RELATIONS, NETWORKS IN THE COMMUNITY
- COMMUNICATION NETWORKS IN THE COMMUNITY
- CULTURAL, RELIGIOUS INFLUENCES
- OTHER EMERGING PUBLIC HEALTH PRIORITIES IN THE COMMUNITY

THIS QUESTION ASKS FOR BRAINSTORMING OF FACTORS IN RELATION TO DESIGN ELEMENTS MENTIONED IN Q1.1, NOT EXHAUSTIVE EXPLANATION OF HOW THE FACTORS INFLUENCE THE PROGRAM. PARTICIPANTS DO **NOT** HAVE TO PROVIDE INFORMATION ON ALL POTENTIAL CONTEXTUAL FACTORS. MAKE SURE THAT YOU OR THE IDI NOTETAKER DOCUMENTS THE FACTORS MENTIONED AND THE CORRESPONDING PROGRAM DESIGN ELEMENT.]

- 1. *Just to confirm, you all have identified the following contextual factors affecting the following elements of the DMPA-SC program in your community.*

[**NOTE TO FACILITATOR:** SUMMARIZE ALOUD, SUCCINCTLY, THE CONTEXTUAL FACTORS MENTIONED IN Q1.2 AND THE CORRESPONDING PROGRAM DESIGN ELEMENTS MENTIONED IN Q1.1]

*Can you rank in the order of most influential to least influential the three most influential contextual factors you discussed moments ago? Explain your justification for ranking these as you do.*

- 1. *Reflecting on the most influential contextual factor, can you elaborate on how it affects the outcomes of the program that you have observed to date?*

[**NOTE TO FACILITATOR:** PROBE SPECIFICALLY FOR PARTICIPANTS’ PERCEPTIONS OF THE INFLUENCE OF FACTORS ON OUTCOMES ON EFFECTIVENESS, FEASIBILITY TO DELIVER THE INTERVENTION THROUGH COMMUNITY STRUCTURES, SUSTAINABILITY OF THE PROGRAM IN THE COMMUNITY].

- 1. *Reflecting on the second most influential contextual factor, can you elaborate on how it affects the outcomes of the program that you have observed to date?*

[**NOTE TO FACILITATOR:** PROBE SPECIFICALLY FOR PARTICIPANTS’ PERCEPTIONS OF THE INFLUENCE OF FACTORS ON OUTCOMES ON EFFECTIVENESS, FEASIBILITY TO DELIVER THE INTERVENTION THROUGH COMMUNITY STRUCTURES, SUSTAINABILITY OF THE PROGRAM IN THE COMMUNITY].

- 1. *Reflecting on the third most influential contextual factor, can you elaborate on how it affects the outcomes of the program that you have observed to date?*

[**NOTE TO FACILITATOR:** PROBE SPECIFICALLY FOR PARTICIPANTS’PERCEPTIONS OF THE INFLUENCE OF FACTORS ON OUTCOMES ON EFFECTIVENESS, FEASIBILITY TO DELIVER THE INTERVENTION THROUGH COMMUNITY STRUCTURES, SUSTAINABILITY OF THE PROGRAM IN THE COMMUNITY].

- 1. *Reflecting on the most influential contextual factor, can you elaborate on how you think it will affect the prospect of scaling up the DMPA-SC program in your to other communities in your district?^[[1]](#footnote-1)^ What do you believe that the program should do in response to the influence of [state the most influential contextual factor]?*

[**NOTE TO FACILITATOR:** FOR THE LATER COMPONENT OF 1.7, PROBE FOR WAYS IN WHICH THE DESIGN ELEMENTS OF THE COMMUNITY PROGRAM MAY NEED TO CHANGE, LEADERSHIP DECISIONS THAT SHOULD BE MADE, MANAGEMENT STRATEGIES FOR MITIGATING THE ADVERSE EFFECTS AND/OR LEVERAGING THE POSITIVE EFFECT OF FACTORS].

- 1. *Reflecting on the second most influential contextual factor, can you elaborate on how you think it will affect the prospect of scaling up the DMPA-SC program to other communities in your district?2 What do you believe that the program should do in response to the influence of [state the most influential contextual factor]?*

[**NOTE TO FACILITATOR:** FOR THE LATER COMPONENT OF 1.8, PROBE FOR WAYS IN WHICH THE DESIGN ELEMENTS OF THE COMMUNITY PROGRAM MAY NEED TO CHANGE, LEADERSHIP DECISIONS THAT SHOULD BE MADE, MANAGEMENT STRATEGIES FOR MITIGATING THE ADVERSE EFFECTS AND/OR LEVERAGING THE POSITIVE EFFECT OF FACTORS].

- 1. *Reflecting on the third most influential contextual factor, can you elaborate on how you think it will affect the prospect of scaling up the DMPA-SC program to other communities in your district?2 What do you believe that the program should do in response to the influence of [state the most influential contextual factor]?*

[**NOTE TO FACILITATOR:** FOR THE LATER COMPONENT OF 1.9, PROBE FOR WAYS IN WHICH THE DESIGN ELEMENTS OF THE COMMUNITY PROGRAM MAY NEED TO CHANGE, LEADERSHIP DECISIONS THAT SHOULD BE MADE, MANAGEMENT STRATEGIES FOR MITIGATING THE ADVERSE EFFECTS AND/OR LEVERAGING THE POSITIVE EFFECT OF FACTORS].

1. **PROGRAMMATIC FEATURES AND IMPLEMENTATION STRATEGIES**
   1. *Reflecting on the progress of the DMPA-SC program in your community to date, can we brainstorm the positive outcomes of it that you have observed to date?*
   2. *Now, can we brainstorm the disappointing outcomes you have observed to date?*
   3. *Let’s all think about the design elements of the DMPA-SC program in your community that you discussed earlier. Which of these are responsible for the positive outcomes you just mentioned? Why do you think these elements are responsible for these outcomes?*

[**NOTE TO FACILITATOR:** SUCCINTLY SUMMARIZE THE PROGRAMMATIC DESIGN ELEMENTS THAT THE KEY INFORMANT DISCUSSED IN Q1.1. PARTICIPANTS CAN MENTION OTHER DESIGN ELEMENTS THAT S/HE DID NOT DISCUSS IN Q1.1 IF THEY WISH TO DO SO. IF NECESSARY TO ELICIT A RESPONSE, YOU CAN STATE THIS TO PARTICIPANTS. PROBE KEY INFORMANT FOR THE FOLLOWING:

- THE PEOPLE THAT WERE RESPONSIBLE FOR IMPLEMENTING THE EFFECTIVE DESIGN ELEMENTS
- HOW THEY IMPLEMENTED THOSE DESIGN ELEMENTS
- WHY THEIR IMPLEMENTATION STRATEGY OR APPROACH WORKED].
  1. *Consider the prospect of scaling up the DMPA-SC program to other communities in your district.2 Which of these program design elements should remain the same during scale up? Which of them should be adapted to enhance scale up?*
  2. *Consider the information we just discussed on the program design elements that you believe must be adapted to enhance scale up. Why should these be adapted? What adaptations and changes should be made to these design elements?*

[**NOTE TO FACILITATOR:** ALLOW PARTICIPANTS TO DESCRIBE HOW THEY THINK DESIGN ELEMENT SHOULD REMAIN AND/OR BE ADAPTED. THEN, PROBE PARTICIPANTS TO REFLECT ON THE INFORMATION THEY JUST SHARED ABOUT IMPLEMENTATION OF THE DESIGN ELEMENTS (E.G. PROBE OF 2.3), AND HOW IMPLEMENTATION STRATEGIES MIGHT BE CONTINUED OR ADAPTED].

- 1. *Let’s recall the design elements of the DMPA-SC program in your community that you all discussed earlier. Which of these are responsible for the disappointing outcomes you just mentioned? Why do you think that these elements are responsible for these outcomes?*

[**NOTE TO FACILITATOR:** PARTICIPANTS CAN MENTION OTHER DESIGN ELEMENTS THAT THEY DID NOT DISCUSS IN Q1.1 IF THEY WISH TO DO SO. IF NECESSARY TO ELICIT A RESPONSE, YOU CAN STATE THIS TO PARTICIPANTS. PROBE PARTICIPANTS FOR THE FOLLOWING:

- THE PEOPLE THAT WERE RESPONSIBLE FOR IMPLEMENTING THE INEFFECTIVE DESIGN ELEMENTS
- HOW THEY IMPLEMENTED THOSE DESIGN ELEMENTS
- WHY THEIR IMPLEMENTATION STRATEGY OR APPROACH WAS NOT EFFECTIVE].
  1. *Consider the prospect of scaling up the DMPA-SC program to other communities in your district.2 Which of these program design elements should be eliminated during scale up? Which of them should be adapted to enhance scale up?*
  2. *Consider the information we just discussed on the program design elements that you believe must be adapted to enhance scale up. Why should these be adapted? What adaptations and changes should be made to these design elements?*

[**NOTE TO FACILITATOR:** ALLOW PARTICIPANTS TO DESCRIBE HOW THEY THINK DESIGN ELEMENT SHOULD REMAIN AND/OR BE ADAPTED. THEN, PROBE PARTICIPANTS TO REFLECT ON THE INFORMATION THEY JUST SHARED ABOUT IMPLEMENTATION OF THE DESIGN ELEMENTS (E.G. PROBE OF 2.6), AND HOW IMPLEMENTATION STRATEGIES MIGHT BE CONTINUED OR ADAPTED].

***[AFTER PARTICIPANTS HAS RESPONDED TO 2.8, READ ALOUD – PARTICIPANTS CAN PARAPHRASE]:*** *That was our final question. Thank you for participating in this discussion. At this time, do you have any further comments or information that you wish to share?*

[**NOTE TO FACILITATOR:** IF PARTICIPANTS SAY YES, ENCOURAGE THEM TO SHARE THEIR COMMENTS. CONTINUE RECORDING THE INTERVIEW.

***[READ ALOUD]:*** *Do you have any questions at this time?*

[**NOTE TO INTERVIEWER:** IF PARTICIPANTS SAY YES, ENCOURAGE THEM TO ASK QUESTIONS. ANSWER THEM TO THE BEST OF YOUR ABILITY. IF YOU CANNOT ANSWER THEM, ENSURE THAT THESE QUESTIONS ARE NOTED AND REPLY THAT YOU WILL DO YOU BEST TO OBTAIN ANSWERS AND REPORT FEEDBACK TO PARTICIPANTS].

***[READ ALOUD]:*** *At this point, I believe we can end this interview. Thank you again.*

**Interviewer or note taker should turn of the digital recorder, record the time at which the interview ends, and depart, leaving with the key informant a business card that includes contact information for the study.**

END TIME OF FGD: ______________________

1. If scale up within the district is not realistic, then suggest that the Key Informant reflects on the prospect of scaling up from her/his district to a neighboring district where the program is not implemented. [↑](#footnote-ref-1)
