# Supplementary material for: Enhancing the Introduction and Scale Up of Self-Administered Injectable Contraception (DMPA-SC) in Health Systems (the EASIER Project): Protocol for Embedded Implementation Research
Source: JMIR Res Protoc. 2023 Aug 23;12:e44222. doi: 10.2196/44222 (PMC10483301; doi:10.2196/44222)
Supplement: Multimedia Appendix 9 [file resprot_v12i1e44222_app9.docx]

**In-depth Interview with DMPA-SC Clients: Reactions to the DMPA-SC Program, Nature of Demand for Family Planning Information and Services, Contraceptive Decision-making.**

**Instructions:**

- This in-depth interview (IDI) is intended to obtain strategic information from key informants that use, and have used, DMPA-SC for self-administration. The information we seek through the IDI include: (a) the nature of and influences on key informants’ demand for family planning (FP) information and services, (b) processes and influences on key informants’ decision-making regarding FP method use, and (c) reactions to the DMPA-SC program.
- Key informants enrolled in these IDI should include women that have initiated self-administration of DMPA-SC in the past 4 months and have decided to either continue DMPA-SC use, discontinue method use, or switch methods.
- Key informants should discuss this information and offer suggestions on the ways in which the DMPA-SC program, and the public sector FP program generally, could be improved in order to better address their demand for FP and perceived needs from and FP program, and address the factors that influence their contraceptive decision-making and use.

| Name of data collector:  ___________________________________________________________________________________  Date:  ___________________________________________________________________________________Country:  ___________________________________________________________________________________Name of Key Informant:  ___________________________________________________________________________________Job Title of Key Informant:  ___________________________________________________________________________________District and community where Key Informant lives: ___________________________________________________________________________________  Date Key Informant initiated DMPA-SC:  ___________________________________________________________________________________  Current contraceptive use status of Key Informant (DMPA-SC user, DMPA-user discontinuer, method-switcher (from DMPA-SC to another method, include the other method): ___________________________________________________________________________________Age of Key Informant  ___________________________________________________________________________________  Marital status of Key Informant  ___________________________________________________________________________________  Number of children of Key Informant  ___________________________________________________________________________________  Highest year of education attained by Key Informant  ___________________________________________________________________________________  Location of the interview:  ___________________________________________________________________________________ |
| --- |

**Introduction:**

**INTERVIEWER SHOULD CONFIRM WITH THE KEY INFORMANT THAT S/HE HAS PERMISSION TO RECORD THE IDI ON A RECORDED DEVICE. IF THE KEY INFORMANT CONSENTS, THE INTERVIEWER CAN START RECORDING NOW. RECORD THE TIME AT WHICH THE INTERVIEW BEGINS.**

START TIME OF IDI: ______________________

***[READ ALOUD – FACILITATORS CAN PARAPHRASE]:*** *Thank you for taking the time to participate in this interview on the DMPA-SC program in [name of community] and your experience as a user of this method. As you are aware, the policies of [name of country] permit the use of DMPA-SC for self-administration. Women who desire the method, can be screened by a healthcare worker and, provided that they are eligible for the method, receive their first injection at the facility together with a re-supply kit of DMPA which they can inject into themselves, sub-cutaneously, at home after their initial injection has expired. As you are aware, this program is being implemented in your community. We are interested in your demand for contraception and perceived needs from a program of family planning information and services (FP). In addition, we would like to understand your preferences concerning contraceptive methods, the process you experience while making decisions on contraceptive use and the factors that influence your decision-making. Finally, we would like to ask you about your reactions to the DMPA-SC program where you accessed that method, your experiences with the method and your decision-making about using the method again. At the end of the interview, you can also make suggestions about ways to improve the FP program overall and the DMPA-SC program specifically.*

**Interview:**

1. **INITIATION OF DMPA-SC: DEMAND FOR CONTRACEPTION, NEEDS FROM AN FP PROGRAM, CONTRACEPTIVE DECISION-MAKING AND SATISFACTION WITH SERVICES**
   1. *I understand that in the past you have used DMPA-SC and self-administered the method. How did you learn about this method?*

- **Probe: social and communication networks where information on the method was transmitted.**
  - **Where did you hear information that encouraged you to use the method?**
  - **Where did you hear information that discouraged you from using the method?**
- **Probe: encouraging and discouraging information can be about DMPA-SC specifically or FP methods in general.**
  1. *What about the method were appealing to you?*
- **Probe: convenience of access, ease of use, duration of protection against unintended pregnancy, privacy, other?**
- **Probe: underlying fertility intentions (desired to space, limit, no desire at the time when she heard about DMPA-SC).**
  1. *What were your concerns?*
- **Probe: safety, management of commodities, side effects, social or spousal concerns, cost.**
  1. *You have mentioned that you [state the fertility intention that motivated them to desire a contraceptive method in 1.2]. Can you explain why this was your preference at that time?*
- **Probe: economic, social, work-related, health-related, spousal, etc. factors that motivated them to prefer to delay or cease childbearing.**
- **Probe: with whom do they discuss their fertility intentions? How do these people influence them?**
  1. *After you heard about DMPA-SC, tell me about the decision-making process you went through to reach the decision to use the method?*
- **Probe: what were your needs from the FP program to help you achieve your fertility intentions?**
- **Probe: Thinking about the appealing aspects (from 1.2), what were the most important to your decision-making.**
- **Probe: Thinking about your concerns (from 1.3), why did you decide to use the method anyway?**
- **Probe: With whom did you discuss the decision? How did they influence you?**
- **Probe: Why did you choose DMPA-SC and not another contraceptive method?**
- **Probe: Any other factors you considered when deciding to use DMPA-SC?**
  1. *Just to confirm, you started to use DMPA-SC on [say date on cover page of interview form, and once the key informant confirms then continue]. Describe your experience initiating this method?*
- **Probe: Where did they receive the service? Who provided the service? What did they like about the experience? What did they not like? What can they recall from the counseling they received?**
  1. *Thinking about the experience you have had since then, has the method met your expectations?*
- **Probe: Review the aspects about DMPA-SC that seemed appealing (question 1.2). Were their beliefs about the method actually true? Why?**
- **Probe: Review the aspects about DMPA-SC that were concerning (question 1.3). Were their concerns about the method actually true? Why?**
- **Probe: To what extent have your FP needs (question 1.4 or needs that emerged after initiating the method) been met and not been met? Why?**
- **Probe: any unanticipated reactions to the method?**
  1. *In general, would you say that you have been satisfied with DMPA-SC so far? Why or why not?*
- **Probe: Continuing information and support regarding side effects; adherence challenges; private space to manage and use the method at home, etc.**

1. **CONTINUATION OF DMPA-SC: CURRENT DEMAND FOR CONTRACEPTION, NEEDS FROM AN FP PROGRAM AND CONTRACEPTIVE DECISION-MAKING**
   1. *At this moment, what are your desires in terms of the timing of future pregnancies?*

- **Probe: If there are changes between the key informants response here at their response in 1.2-1.3, ask her to explain the reason for this change.**
- **Probe: economic, social, work-related, health-related, spousal, etc. factors that motivated them to prefer to delay or cease childbearing.**
- **Probe: with whom do they discuss their fertility intentions? How do these people influence them?**
  1. *It is my understanding that you have chosen to [state whether the key informant chose to continue, discontinue or switch methods. Once the key informant confirms, then continue]. Can you tell me about the decision-making process you went through to reach this decision?*
- **Probe: social pressures, heard new information about DMPA-SC or other FP methods, experiences with side effects, spousal discord, cost, desire for another method, change in fertility desires, forgot to re-inject?**
- **Probe: based on your experience with DMPA-SC, do you feel that this service can meet your current needs for contraception? Why or why not?**
- **Probe: based on your experience with DMPA-SC, what else is required to meet your needs:**
  - **Other contraceptive methods, more information and support after initiation, more privacy at home, reminder about timing about re-injection, help paying for the method, more support from community to create an enabling environment for FP use, help dealing with side effects, support with spousal communication.**
- **Probe: With whom did you consult while making this decision? How did they help you?**

1. **WAYS THAT THE FP PROGRAM CAN BETTER MEET WOMEN’S DEMAND FOR CONTRACEPTION AND RELATED NEEDS.**
   1. *Reflecting on your experiences and relationships with other women in your community, explain what you believe are women’s needs for FP information and services, including contraceptive methods.*

- **Probe: access to a wide range of contraceptive methods, information, education and communication, community support, spousal communication and support, clear and thorough counseling, etc.**
  1. *On a scale of 1 to 5, where 1 equals very poorly and 5 equals very well, please rate how well you think the FP program that you have accessed meets women’s needs for FP information and services?*
- **Probe: Encourage the key informant to consider the entire FP program and not only DMPA-SC services she has received.**
- **Probe: Ask the key informant to describe what they perceive as strengths and weaknesses of the FP program.**
  1. *On a scale of 1 to 5, where 1 equals very poorly and 5 equals very well, please rate how well you think the DMPA-SC program that you have accessed meets women’s needs for FP information and services?*
- **Probe: Ask the key informant to describe what they perceive as strengths and weaknesses of the FP program.**

***[AFTER THE KEY INFORMANT HAS RESPONDED TO 3.3, READ ALOUD – INTERVIEWERS CAN PARAPHRASE]:*** *That was our final question. Thank you for participating in this interview. At this time, do you have any further comments or information that you wish to share?*

[**NOTE TO INTERVIEWER:** IF THE KEY INFORMANT SAYS YES, ENCOURAGE HIM/HER TO SHARE HIS/HER COMMENTS. CONTINUE RECORDING THE INTERVIEW.

***[READ ALOUD]:*** *Do you have any questions at this time?*

[**NOTE TO INTERVIEWER:** IF THE KEY INFORMANT SAYS YES, ENCOURAGE HIM/HER TO ASK QUESTIONS. ANSWER THEM TO THE BEST OF YOUR ABILITY. IF YOU CANNOT ANSWER THEM, ENSURE THAT THESE QUESTIONS ARE NOTED AND REPLY THAT YOU WILL DO YOU BEST TO OBTAIN ANSWERS AND REPORT FEEDBCK TO THE KEY INFORMANT].

***[READ ALOUD]:*** *At this point, I believe we can end this interview. Thank you again.*

**Interviewer or note taker should turn of the digital recorder, record the time at which the interview ends, and depart, leaving with the key informant a business card that includes contact information for the study.**

END TIME OF IDI: ______________________
