# Supplementary material for: Enhancing the Introduction and Scale Up of Self-Administered Injectable Contraception (DMPA-SC) in Health Systems (the EASIER Project): Protocol for Embedded Implementation Research
Source: JMIR Res Protoc. 2023 Aug 23;12:e44222. doi: 10.2196/44222 (PMC10483301; doi:10.2196/44222)
Supplement: Multimedia Appendix 10 [file resprot_v12i1e44222_app10.docx]

**Community-level Focus Group Discussion: Reactions to the DMPA-SC Program, Nature of Community Demand for Family Planning Information and Services and DMPA-SC Services.**

**Instructions:**

- This focus group discussion (FGD) is intended to obtain strategic information from key informants on: (a) community reactions to the activities of the self-administered DMPA-SC program (b) the extent to which the program is acceptable vis-à-vis community, social and cultural characteristics and (c) the nature of communities’ demand for family planning information and services, generally, and DMPA-SC in particular.
- Focus groups should comprise of participants that are members of communities where the DMPA-SC program is implemented. These include: political leaders (e.g. village governments, health committees), opinion leaders (e.g. women’s groups, youth groups), users and potential users of DMPA-SC, the spouses of uses and potential users of DMPA-SC, etc. In all groups, participants should describe their reactions to the DMPA-SC program in their community, the aspects they think conform and clash with community, social, and cultural characteristics (e.g. gender relations, religious norms, social relations, communication and social networks). In addition, they should discuss their perceptions of why the community desires, and resists, family planning information and services, and how the DMPA-SC program addresses this demand and resistance.
- They should discuss this information and offer suggestions on the ways in which the program could be improved in order to better conform to the community, social and cultural characteristics and the nature of the communities’ demand for information and services they emphasize in the discussion.
- FGD should be segmented according to the participant categories discussed above, ensuring that groups comprise of members of the same gender, age group and any other particular characteristics in order to ensure that all participants can contribute pertinently and comfortably to the lines of questioning and commentary put forth by their peers.
- The FGD should have between 6-8 participants.
- The FGD should be facilitated by two facilitators. One facilitator should lead the discussion and take notes. The second should also take notes and also, where necessary, record salient points from the discussion on flip charts.

| Name of data collector:  ___________________________________________________________________________________________  Date: ___________________________________________________________________________________________Country, District and Community:  ___________________________________________________________________________________________  Location of the interview:  ___________________________________________________________________________________________  Focus group discussion type (i.e. male opinion leaders, female opinions, women age 20-34, women age 34 and above, spouses of DMPA-SC users, adolescents and youth)  ___________________________________________________________________________________________  Name of FGD Member 1:  ___________________________________________________________________________________________  Job Title FGD Member 1:  **___________________________________________________________________________________________**  Name of FGD Member 2:  ___________________________________________________________________________________________  Job Title FGD Member 2:  ___________________________________________________________________________________________  Name of FGD Member 3:  ___________________________________________________________________________________________  Job Title FGD Member 3:  ___________________________________________________________________________________________  Name of FGD Member 4:  ___________________________________________________________________________________________  Job Title FGD Member 4:  ___________________________________________________________________________________________  Name of FGD Member 5:  ___________________________________________________________________________________________  Job Title FGD Member 5:  ___________________________________________________________________________________________  Name of FGD Member 6:  ___________________________________________________________________________________________  Job Title FGD Member 6:  ___________________________________________________________________________________________ |
| --- |

**Introduction:**

**FACILITATOR SHOULD CONFIRM WITH THE FGD PARTICIPANTS THAT S/HE HAS PERMISSION TO RECORD THE FGD ON A RECORDED DEVICE. IF THE PARTICIPANTS CONSENT, THE INTERVIEWER CAN START RECORDING NOW. RECORD THE TIME AT WHICH THE INTERVIEW BEGINS.**

START TIME OF FGD: ______________________

***[READ ALOUD – FACILITATORS CAN PARAPHRASE]:*** *Thank you for taking the time to participate in this focus group discussion on the DMPA-SC program in [name of community]. As you are aware, the policies of [name of country] permit the use of DMPA-SC for self-administration. Women who desire the method, can be screened by a healthcare worker and, provided that they are eligible for the method, receive their first injection at the facility together with a re-supply kit of DMPA which they can inject into themselves, sub-cutaneously, at home after their initial injection has expired. As you are aware, this program is being implemented in your community. We are interested in your perceptions of the DMPA-SC program, as it is being carried out in your community. In particular, we are interested in your reactions to the program, the extent to which the program is acceptable vis-à-vis community, social and cultural norms, attitudes, practices, beliefs in your community. Also, we would like to know your perceptions of why the community desires, and resists, family planning information and services, and how the DMPA-SC program in your community addresses this demand and resistance. Finally, we would like your suggestions on how program could be improved in order to better conform to the community, social and cultural characteristics and the nature of your communities’ demand for family planning information and services.*

**Discussion:**

1. **NATURE OF COMMUNITY DEMAND FOR FAMILY PLANNING (FP) INFORMATION AND SERVICES**
   1. *Let us think about FP information and services in general. What does this mean to all of you? What are the different elements of family planning and services based on your understandings?*

- **Probe for individuals’ and group’s understandings and conceptualization of what FP information and services is to them broadly and what the service components are.**
- **If necessary, provide clarification of components of FP information and services, e.g. information, education and communication; counseling; contraceptive methods; service delivery modalities like community-based distribution; ways to make FP info and services affordable, etc.**
- **List the elements that are discussed on a flip chart and summarize these as elements of a family planning (FP) program.**
- **Probe for any additional elements of a FP program that were not discussed.**
  1. *Thinking about what we just discussed, what are most important elements of an FP program?*
- **Probe for elements such as models that made FP accessible to people, individualized counseling and written information materials, preferred methods.**
- **Probe for personal beliefs about the most important elements of an FP program;**
- **When you exhaust individual beliefs, ask about what is most important to their household and families.**
  1. *Why are these elements important? Can you describe your perceptions of the benefits of an FP program?*
- **If necessary, probe for effects of FP on rearing a family, health of women, timing and spacing of childbearing.**
- **Probe for the group to share their fertility desires and how they would like an FP program to help them achieve them.**
  1. *What elements of an FP program that present difficulties or challenges to those who wish to access it?*
- **Probe for difficulties that stir difficulties such as geographic distance, lack of knowledge and education, cost/expense of methods, social stigma and rumors, spousal discord, gender relations, religious issues, health and fertility concerns.**
  1. *Why do these elements present difficulties? Can you describe experiences you have had dealing with these difficulties?*
  2. *Can we rank and discuss the biggest needs and desires of the group concerning family planning information and services?*
- **Probe for needs and desires in terms of maximizing the benefit and expanding the important elements of family planning information and services**
- **Probe for needs and desires in terms of addressing the difficulties and challenges presented by family planning information and services.**
- **Generate 6-8 of the biggest needs and desires of the focus group and have them rank these on a flip chart.**

1. **COMMUNITY, SOCIAL AND CULTURAL NORMS, ATTITUDES AND PRACTICES AND FP PROGRAMS**
   1. *Let us think about your community now. Can we brainstorm the characteristics and structures of the community that influence if people can meet their FP needs and achieve their FP desires? Just to clarify, we can discuss things that help or hinder people from meeting their FP needs and desires.*

- **Probe for structures such as community governance structures, community health groups and committees, influence of opinion leaders, religious organizations, economic and work arrangements.**
  - - **How do such factors shape norms, attitudes, decision-making and behaviors about FP?**
- **Probe for social relations about gender, spousal relations, adolescence and youth, stigma, social networks, cultural beliefs, how people communicate and share information.**
  - - **How do such factors shape norms, attitudes, decision-making and behaviors about FP?**
  1. *Let us think about these characteristics and structures of the community that we just discussed and the FP program that is available to you. What are the aspects of the FP program work well with these community realities? Which do not work well?*
- **Note that we are not just talking about the DMPA-SC program now, we mean the entire FP program.**
- **Review the elements of the FP program discussed during section 1 of the FGD and the responses given to question 2.1.**
- **Probe: what important elements of the FP program are not working well given the factors raised in 2.1? What difficulties does the FP program struggle to address because of the factors raised in 2.1.**
  1. *Let us think about the challenges that we have discussed between the FP program and your community. What can the community do to address these challenges?*
  2. *Let us think about those challenges. What can the public health system’s FP program do to address those challenges?*

1. **THE COMMUNITY AND THE DMPA-SC PROGRAM**

*Let us think about the program about DMPA-SC, which involves women receiving family planning supplies from the FP program and administering the FP injection to themselves at home.*

- 1. *Describe for us how this program works? Both at the level of healthcare facilities and within the community?*
- **Probe for roles of healthcare workers and staff at facilities, roles of members of the community (leaders, committees, opinion leaders, FP users, community health workers, volunteers).**
  1. *What are your reactions to this program? What do you like about it?*
- **Probe: review the needs and desires from an FP program that the group discussed in section 1. How does the DMPA-SC program satisfy those needs and desires?**
  1. *What do you not like about it?*
- **Probe: review the needs and desires from an FP program that the group discussed in section 1. How does the DMPA-SC program not satisfy those needs and desires?**
  1. *Let us think about all of the community characteristics and structures we discussion in the previous section. Given those realities about your community, what elements of the DMPA-SC program do you think are appropriate?*
- **Probe: why are these elements appropriate? Ask for examples.**
  1. *Given those realities about your community, what are the elements of the DMPA-SC program think are not appropriate?*
- **Probe: why are these elements not appropriate? Ask for examples.**
  1. *What should be done to improve the DMPA-SC program?*
- **Probe: What can the community do? What can the health system do?**
- **Probe: for both community and health system, what can be done to improve how the program satisfies needs and desires for an FP program./**
- **Probe: for both community and health system, what can be done to make the program more appropriate?**
  1. *Rank according to priority to 5 most important things that can be done to improve the DMPA-SC program.*
- **Facilitate a discussion where participants agree on how to rank the points mentioned in 3.6 by priority.**

***[AFTER PARTICIPANTS HAS RESPONDED TO 3.7, READ ALOUD – PARTICIPANTS CAN PARAPHRASE]:*** *That was our final question. Thank you for participating in this discussion. At this time, do you have any further comments or information that you wish to share?*

[**NOTE TO FACILITATOR:** IF PARTICIPANTS SAY YES, ENCOURAGE THEM TO SHARE THEIR COMMENTS. CONTINUE RECORDING THE INTERVIEW.

***[READ ALOUD]:*** *Do you have any questions at this time?*

[**NOTE TO INTERVIEWER:** IF PARTICIPANTS SAY YES, ENCOURAGE THEM TO ASK QUESTIONS. ANSWER THEM TO THE BEST OF YOUR ABILITY. IF YOU CANNOT ANSWER THEM, ENSURE THAT THESE QUESTIONS ARE NOTED AND REPLY THAT YOU WILL DO YOU BEST TO OBTAIN ANSWERS AND REPORT FEEDBACK TO PARTICIPANTS].

***[READ ALOUD]:*** *At this point, I believe we can end this interview. Thank you again.*

**Interviewer or note taker should turn of the digital recorder, record the time at which the interview ends, and depart, leaving with the key informant a business card that includes contact information for the study.**

END TIME OF FGD: ______________________
